# Design, Synthesis, Characterization, and Analysis of Antimicrobial Property of Novel Benzophenone Fused Azetidinone Derivatives through In Vitro and In Silico Approach

**DOI:** 10.3390/cimb45010007

**Published:** 2022-12-23

**Authors:** Lakshmi Ranganatha Venkataravanappa, Mahima Jyothi, Hussien Ahmed Khamees, Ekaterina Silina, Victor Stupin, Raghu Ram Achar, Mohammed Al-Ghorbani, Shaukath Ara Khanum

**Affiliations:** 1 Department of Chemistry, The National Institute of Engineering, Mysuru 570008, Karnataka, India; 2 Department of Chemistry, Yuvaraja’s College (Autonomous), University of Mysore, Mysuru 570005, Karnataka, India; 3 Department of Medical Science, Community College-Abs, Hajjah ABS-00967, Yemen; 4 Department of Surgery, Pirogov Russian National Research Medical University, 117997 Moscow, Russia; 5 Institute of Biodesign and Modeling of Complex Systems, I.M. Sechenov First Moscow State Medical University (Sechenov University), 119435 Moscow, Russia; 6 Division of Biochemistry, School of Life Sciences, JSS Academy of Higher Education & Research, Mysuru 570015, Karnataka, India; 7 Department of Chemistry, College of Science and Arts, Ulla, Taibah University, Medina 41477, Saudi Arabia; 8 Department of Chemistry, College of Education, Thamar University, Thamar 425897, Yemen

**Keywords:** 2-Azetidinone, benzophenone, antimicrobial, in vitro, in silico, molecular docking simulations

## Abstract

A sequence of novel 2-(4-benzoyl-2-methyl-phenoxy)-N-(3-chloro-2-oxo-4-phenyl-azetidin-1-yl)-acetamide analogues **9(a–n)** were synthesized by multistep synthesis. The newly synthesized compounds were well characterized, and their antimicrobial activities were carried out by disc diffusion and broth dilution methods. Further, all the novel series of compounds (**9a**–**n**), were tested against a variety of bacterial and fungal strains in comparison to *Ketoconazole, Chloramphenicol,* and *Amoxicillin* as standard drugs, respectively. Compounds **9a**, **9e**, and **9g** as a lead molecule demonstrated a good inhibition against tested strains. Further, molecular docking studies have been performed for the potent compounds to check the three-dimensional geometrical view of the ligand binding to the targeted proteins.

## 1. Introduction

For the past several years the emergence of organisms resistant to almost all the classes of antimicrobial agents has become a serious public health concern [1,2]. The discovery and designing of new anti-microbial drugs in the pursuit of better treatment have been the main goal for scientists. In recent decades, problems of multi-drug resistant microorganisms have reached an alarming level in many countries around the world [3]. WHO has declared that AMR (antimicrobial resistance) is one of the top ten global public health threats facing humanity. Yet the number of people facing antibiotic resistance in the United States is still too high and also more than 2.8 million antibiotic-resistant infections occur in the United States each year, in addition, more than 35,000 people die as a result. Further, nearly 223,900 people in the United States required hospital care for Clostridioides difficile and at least 12,800 people died in 2017 [4]. Resistance to several anti-microbial agents (β-lactamase antibiotics, macrolides, quinolones and vancomycin) has been reported; also, a number of current clinical reports describe the increasing occurrence of Methicillin resistant Staphylococcus aureus (MRSA), Drug-resistant Streptococcus pneumoniae (S. pneumoniae), Carbapenem-resistant Enterobacteriaceae (CRE), Erythromycin-resistant group A Streptococcus, and Clindamycin-resistant group B Streptococcus, which is the most disturbing cause of serious infections in developed countries [5,6]. Even in past decades, it has been a challenging increase in the frequency of systematic fungal infection in man. The first orally active antifungal agent that was effective against a broad collection of systematic and superficial fungal infections was ketoconazole [7]. Further, a few azoles antifungal agents viz., itraconazole [8], fluconazole [9], voriconazole [10], ravuconazole [11] etc., and glucan synthesis inhibitor caspofungin [12] have been introduced to the clinic. Antibiotics are one of our most vital weapons in fighting bacterial infections and have significantly benefited the quality of health-related human life since their introduction. However, these health benefits are under threat as many commonly used antibiotics have become less and less effective against certain illnesses, not only because many of them produce toxic reactions but also due to the emergence of drug resistant bacteria. Infections caused by these microorganisms pose a serious challenge to the medical community and the need for effective therapy has led to the search for novel antimicrobial drugs with lesser resistance [13].

2-Azetidinone is a four membered heterocyclic amide, commonly known as β-lactam, a well-known compound among organic and medicinal chemists for their structural feature of a number of broad-spectrum β-lactam antibiotics, including penicillins, cephalosporins, carbapenems, nocardicin, monobactams, clavulanic acid, sulbactam, and tazobactam, which have been extensively used as chemotherapeutic agents to treat bacterial infections and microbial diseases [14,15,16,17,18,19,20,21,22,23,24]. Azetidinones are one of the important class of compounds possessing a wide range of biological activities [25,26,27,28,29,30]. Aside from their biological activities, the importance of β -lactams as synthetic intermediates have been widely recognized in organic synthesis [31], for example in the semi synthesis of Taxol [32]. Like azetidinone, benzophenone analogues also showed extensive evidence to establish the efficiency as anticancer [33,34,35], anti-inflammatory [36] and antimicrobial agents [37,38]. In the light of these facts, here we hybridized benzophenone with azetidinone moiety. Moreover, the antimicrobial and antifungal activities of the synthesized compounds have been predicted virtually by using in silico docking simulations. The antimicrobial target in docking studies was selected based on the literature survey which reported β-lactam, an excellent inhibitor of transpeptidases, making it a potent antibiotic class used to treat bacterial infections and microbial diseases [14,15,16,17,18,19,20,21,22,23,24]. Moreover, the targets of antifungals have been also chosen based on the literature survey that reported azetidinone as good inhibitor of CYP51 and other P450s enzymes in fungi. The inhibition of these enzymes causes the accumulation of membrane-disrupting methylated sterol precursors of ergosterol, preventing fungal growth, similar to the original inhibitor, Ketoconazole, which has the mechanism of inhibition of the fungal 14-alpha-demethylase enzyme and also used as standard drug in vitro studies of the current work.

Based on the findings and docking-simulated interaction we have made an emphasis that among the newly synthesized series, the compounds **9a**, **9e**, and **9g** emerged as potent antimicrobial agents which can be used as a potential drug in the near future.

## 2. Materials and Methods

All solvents and reagents were purchased from Sigma Aldrich Chemicals Pvt Ltd. India, Melting points were determined on an electrically heated VMP-III melting point apparatus. The FT-IR spectra were recorded using KBr discs and Nujol on FT-IR Jasco 4100 infrared spectrophotometer. ^1^H NMR spectra were recorded using Bruker DRX 400 spectrometer at 400 MHz with TMS as an internal standard. Mass spectra were recorded on LC-MS (API-4000) mass spectrometer. Further elemental analysis of the compounds was performed on a Perkin Elmer 2400 elemental analyzer.

### 2.1. Chemistry

#### 2.1.1. General Procedure for the Preparation of Phenyl Benzoates **3(a–b)**

Substituted benzoates **3(a–b)** were synthesized by benzoylation of *o*-cresol (**1,** 0.001 mol) with corresponding benzoyl chlorides **2(a–b)**, 0.001 mol) using 10% sodium hydroxide solution. The reaction mixture was stirred for 2–3 h at 0 °C and monitored by TLC using 4:1 n-hexane: ethyl acetate solvent mixture. After completion of the reaction, the organic layer was extracted with ether (3 × 15 mL). Ether layer was washed with 10% sodium hydroxide solution (3 × 30 mL) followed by water (3 × 25 mL) and then dried over anhydrous sodium sulphate and evaporated. The resulting solid was crystallized in ethanol to afford desired compounds **3(a–b)** in a pure state. Refer to Supplementary File for characterization data.

#### 2.1.2. General Procedure for the Preparation of Substituted 4-Hydroxy Benzophenones **4(a–b)**

Substituted 4-hydroxy benzophenones **4(a–b)** were synthesized by Fries rearrangement. Compounds **3(a–b)** (0.001 mol) was treated with anhydrous aluminium chloride (0.002 mol) as a catalyst and heated at 150–170 °C temperature under neat condition for about 2–3 h. Then the reaction mixture was cooled to room temperature quenched with 6N HCl in the presence of ice-cold water and stirred for about 2–3 h. The solid was filtered and recrystallized from ethanol to obtain compounds **4(a–b**) in pure form.

#### 2.1.3. General Procedure for the Preparation of Ethyl 2-(4-Benzoyl-2-Methylphenoxy) Acetates **5(a–b)**

Compounds **5(a–b)** were obtained by refluxing a mixture of compounds **4(a–b)** (0.013 mol) and ethyl chloroacetate (0.026 mol) in dry acetone (35 mL) and in presence of weak base anhydrous potassium carbonate (0.019 mol) for 8–9 h. The reaction mixture was cooled, and the solvent was removed by distillation. The residual mass was triturated with cold water to remove potassium carbonate and extracted with ether (3 × 50 mL). The ether layer was washed with 10% sodium hydroxide solution (3 × 50 mL) followed by water (3 × 30 mL) and then dried over anhydrous sodium sulphate and evaporated to dryness to obtain crude solid, which on recrystallization from ethanol afforded compounds **5(a–b)** in a pure state. 

#### 2.1.4. General Procedure for the Preparation of Substituted 2-(4-Benzoyl-2-Methylphenoxy) Acetohydrazides **6(a–b)**

To compounds **5(a–b)** (0.01 mol) in ethanol (10 mL) 99% hydrazine hydrate (0.01 mol) was added dropwise and continuously stirred for 2 h at room temperature to achieve compounds **6(a–b**) as a white solid. The solid was recrystallized with methanol to obtain pure product **6(a–b)**.

#### 2.1.5. General Procedure for the Preparation of Substituted 2-(4-Benzoyl-2-Methylphenoxy)-N-Benzylideneacetohydrazide **8(a–n)**

To a solution of compounds **6(a–b)** (0.01 mol) in absolute ethanol (50 mL), a catalytic amount of acetic acid and an equimolecular number of corresponding aldehydes **7(a–g)** was added. The reaction mixture was refluxed for 8–10 h and after completion of the reaction, the reaction mixture was cooled to room temperature, poured into crushed ice, filtered, washed, dried, and recrystallized from acetonitrile to yield compounds **8(a–n)** in a good yield. 

#### 2.1.6. General Procedure for the Preparation of 2-(4-benzoyl-2-methyl-phenoxy)-N-(3-chloro-2-oxo-4-phenyl-azetidin-1-yl)-Acetamides **9(a–n)**

The compounds (**8(a–n)** (0.01 mol) and triethylamine (0.01 mol) were dissolved in dioxane (50 mL), cooled, and stirred. To this well-stirred cold solution, chloroacetyl chloride (0.01 mmol) was added drop wise within a period of 20 min. The reaction mixture was then stirred for an additional 3 h and left at room temperature for 48 h. The resultant mixture was concentrated, cooled, poured into ice cold water, filtered, and then dried. The product thus obtained was purified by column chromatography over silica gel using 30% ethyl acetate: 70% benzene as an eluent. Recrystallization was done from suitable solvent which gave 2-azetidinones derivatives **9(a–n)**.

### 2.2. Pharmacology

#### 2.2.1. In Vitro Antibacterial and Antifungal Activity

##### Antimicrobial Testing

The compounds **9(a–n)** were dissolved in DMSO at different concentrations 12.5, 25, 50 and 100 μg/well. Bacterial strains *Staphylococcus aureus, Bacillus subtilis, Klebsiella pneumonia, Pseudomonas aeruginosa,* and fungi *Aspergillus niger* and *Pencillium chrysogenum* were obtained from the Department of Microbiology, Manasagangotri, Mysore, India.

#### 2.2.2. Antimicrobial and Antifungal Assays

The in vitro antimicrobial studies were carried out by the agar wells diffusion method against test organisms [39,40]. Nutrient broth (NB) plates were swabbed with 24 h old broth culture (100 μL) of test bacteria. Using the sterile cork borer, wells (6 mm) were made into each Petri plate. The compounds were dissolved in DMSO of 5 mg/mL and from this 2.5, 5, 10, and 20 μL (12.5, 25, 50, 100 μg/mL) were added into the wells by using sterile pipettes. Simultaneously the standard antibiotics, *Chloramphenicol* and *Amoxicillin* for antibacterial activity and *Ketoconazole* for antifungal activity (as positive control) were tested against the pathogens. The samples were dissolved in DMSO which showed no zone of inhibition acts as a negative control. The plates were incubated at 37 °C for 24 h for bacteria and at 28 °C for 48 h for fungi. After appropriate incubation, the diameter of the zone of inhibition of each well was measured. Duplicates were maintained and the average values were calculated for eventual antimicrobial activity. A broth dilution test was used to determine the Minimum Inhibitory Concentration (MIC) of the above-mentioned samples [41,42]. The freshly prepared nutrient broth was used as diluents. The 24 h old culture of the test bacteria *S. aureus, B.subtilis, P. aeruginosa and K. pneumoniae* and the test fungi *A. Niger* and *P. Chrysogenum* were diluted 100 folds in nutrient broth (100 μL bacterial cultures in 10 mL NB). The stock solution of the synthesized compounds was prepared in DMSO by dissolving 5 mg of the compound in 1 mL of DMSO. Increasing concentrations of the test samples (1.25, 2.5, 5, 10, 20, 40 μL of a stock solution containing 6.25, 12.5, 25, 50, 100, 200 μg of the compounds) were added to the test tubes containing the bacterial and fungal cultures. All the tubes were incubated at 37 °C for 24 h for bacteria and at 28 °C for 48 h for fungi. The tubes were examined for visible turbidity and using NB as control. Control without test samples and with solvent was assayed simultaneously. The lowest concentration that inhibited the visible growth of the tested organisms was recorded as MIC. To determine the Minimum Bactericidal Concentration (MBC) [43] and Minimum Fungicidal Concentration (MFC) [44] for each set of test tubes in the MIC determination, a loopful of broth was collected from those tubes which did not show any growth and inoculated on sterile nutrient broth (for bacteria) and PDA (for fungi) by streaking. Plates inoculated with bacteria and fungi were incubated at 37 °C for 24 h and at 28 °C for 48 h, respectively. After incubation, the lowest concentration was noted as MBC (for bacteria) or MFC (for fungi) at which no visible growth was observed.

### 2.3. Docking Simulation (Methodology)

Docking simulations were carried out by using AutoDock Tools version 4 (ADT4) [45]. The structure data of target proteins (pdb ID: 5E1G) for antibacterial and (pdb ID: 3LD6) for antifungal were downloaded from RCSB PDB website (http://www.rcsb.org/pdb/ accessed on 5 October 2022). They were selected based on the literature survey, which reported β-lactam and azetidinone as potent inhibitors of microbial and fungal protein targets that could be utilized to devolve the antimicrobial and antifungal therapies. Furthermore, the skeleton of our synthesized compounds shared substructure features with the target’s cocrystal ligand. Hence, based on the on the literature data and shared substructure, we have chosen 5E1G and 3LD6 proteins as targets of antibacterial and antifungal for in silico docking studies with the compounds.

Earlier to docking simulations, all co-crystal ligands, ions, and water molecules have been detached from the proteins. Moreover, charge neutralization, polar hydrogens setting, and rotatable bonds were processed by ADT4. On the other hand, Chem Draw Ultra 12.0 was adopted to construct the ligands and minimized the energy by using MM2 force field and then saving them in pdb format. In silico docking studies for all ligands against proteins were executed with the Lamarckian Genetic Algorithm (LGA) model [46] that was extensively employed to predict the binding modes and conformations [47,48]. The grid map was cantered at the active site pocket of the proteins with grid box dimensions of 120 × 120 × 120 Å^3^ points and grid-point spacing of 0.425 Å. Amongst all ligand-receptor docking results, the ligands **9a, 9e**, and **9g** showed a significant affinity score with remarkable hydrogen bonds. Table 1 lists the conformation details of ten docking results for **9a, 9e**, and **9g** compounds including the binding energy, hydrogen bonds interactions, and ligand efficiency (LE), that are calculated as the ratio of Gibbs free energy of binding (G) to the number of non-hydrogen atoms of the compound (i.e., a result of dividing the Gibbs free energy of binding (G) by the number of heavy atoms) [49], which can be given mathematically as LE = (G)/N. 

Using the thermodynamic equation for Gibbs free energy, Δ*G* = −RTlnK_i*,*_ and substituting IC_50_ for *K*_i_ (a less rigorous approximation), ligand efficiency can be calculated as follows [50]: LE = 1.4(pIC_50_)/*N*

Besides ADT4, we have also utilized BIOVIA [51] and PyMOL [52] software to visualize and present the conformations.

## 3. Results and Discussion

### 3.1. Structure-Based Design

**Figure 1:**
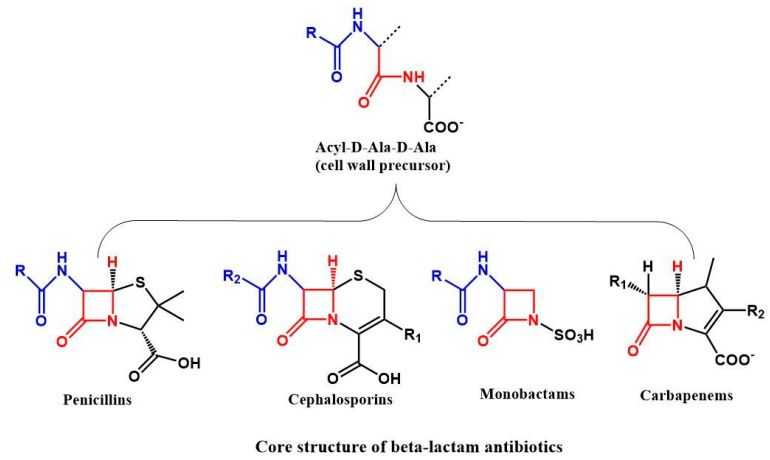
β-Lactam antibiotics.

**Figure 2:**
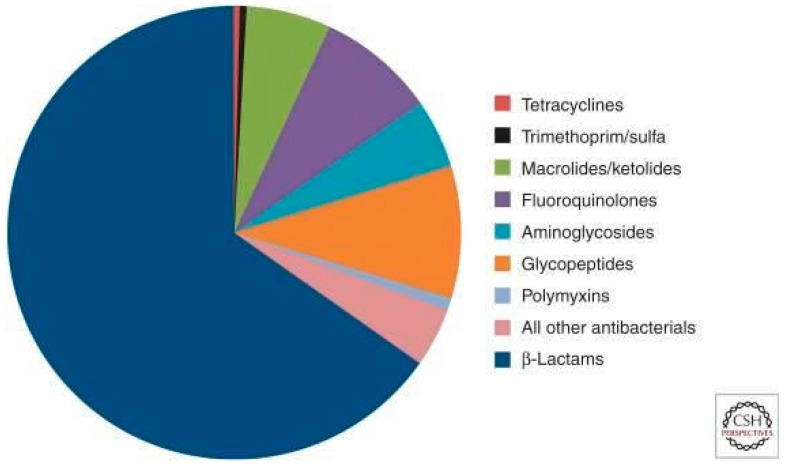
Proportion of prescriptions in the United States for injectable antibiotics by class for years 2004–2014.

**Figure 3:**
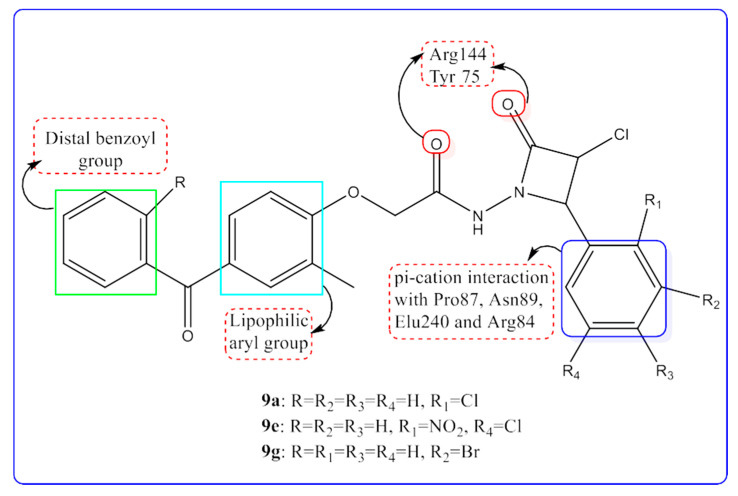
Design strategy.

An adequate literature study was carried out to reveal the importance of four membered nitrogen containing heterocyclic compound, in particularly azetidinone and also about benzophenone analogues. The study showed that researchers reported excellent antimicrobial activity of azetidinone analogues [53,54]. A few benzophenone analogues reported by our group are also promising antimicrobial agents [2,55,56,57,58]. In addition, drugs such as penicillins, cephalosporins, carbapenems, monobactams consist of the β-lactam ring (Figure 1), and they are renowned antimicrobial drugs. β-Lactam antibiotics are currently the most used class of antibacterial agents in the infectious disease armamentarium. As shown in (Figure 2), β-lactams account for 65% of all prescriptions for injectable antibiotics in the United States. Nevertheless, the title compounds contain essential pharmacophoric elements that are essential for a molecule to exhibit antimicrobial activity such as a β-lactam ring, distal benzoyl group, lipophilic aryl group, and the donor nitrogen atom of acetamide bridge. Furthermore, the carbonyl oxygen of lactam ring forms hydrogen bonding interaction with Arg144 and Tyr75 and even amide oxygen showed hydrogen bonding interaction with Arg144. The substituted phenyl ring exhibited pi-cation interaction with Pro87, Asn89, Elu240 and Arg84 residues. Based on these points we designed new analogues containing N-CO and other pharmacophores necessary to show antimicrobial performance (Figure 3).

### 3.2. Chemistry

**Scheme 1:**
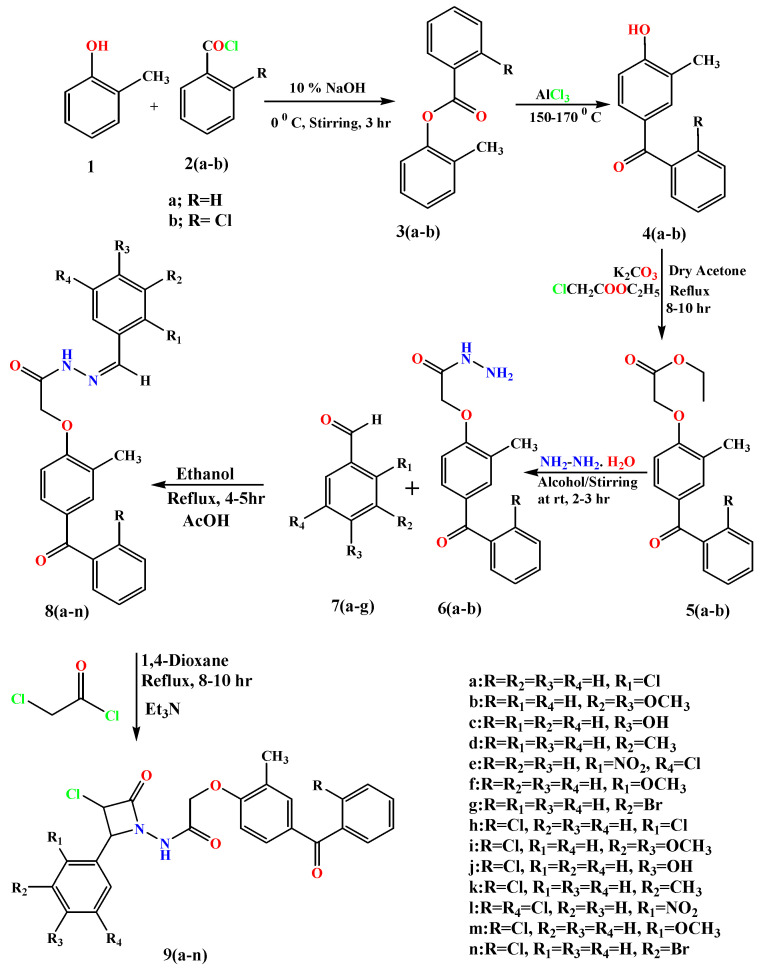
Synthesis of 2-(4-Benzoyl-2-methyl-phenoxy)-N-(3-chloro-2-oxo-4-phenyl-azetidin-1-yl)-acetamide analogues (9a–n).

The reaction sequence for different title compounds **9(a–n)** was accomplished by a synthetic procedure as shown in (Scheme 1). All the synthesized compounds were characterized by IR, NMR, and mass spectral data. The starting materials substituted phenyl benzoate analogues (**3a–b)** were synthesized by benzoylation of *o*-cresol (**1**) with corresponding benzoyl chlorides **2(a–b)** using 10% sodium hydroxide solution. Fries rearrangement of compounds **3(a–b)**, was carried out under neat conditions, using anhydrous aluminium chloride as a catalyst to afford hydroxy benzophenones **4(a–b)**. The compounds **4(a–b)** on etherification with ethyl chloroacetate using dry acetone as a solvent gave substituted ethyl 2-(4-benzoylphenoxy) acetates **5(a–b)**. The compounds **5(a–b)** in ethanol were treated with hydrazine hydrate dropwise, with continuous stirring for two hours to achieve substituted 4-benzoyl-phenoxy aceto hydrazides **6(a–b)**. Further, the compounds **6(a–b)** in absolute ethanol were treated with substituted aldehydes **7(a–g)** with a catalytic amount of acetic acid and refluxed for 8–10 h to obtain substituted 2-(4-benzoyl-2-methylphenoxy)-N-(2-benzylidene) acetohydrazides **8(a–n)**. Finally, the compounds **8(a–n)** and triethyl amine as catalyst in dioxane were cooled and stirred. To this well-stirred cold solution, chloroacetyl chloride was added dropwise within a period of 20 min and stirring was continued for an additional 3 h to furnish the title compounds substituted 2-(4-benzoyl-2-methyl-phenoxy)-N-(3-chloro-2-oxo-4-phenyl-azetidin-1-yl)-acetamides **9(a–n)**. Among **3(a–b)** the spectrum of compound (**3a**), is selected as a representative example. The formation of this compound was confirmed by the appearance of the carbonyl stretching band for the ester group at 1715 cm^−1^ in the IR spectrum and the appearance of nine aromatic protons between 7.0 and 7.8 ppm in the proton NMR spectrum. The mass spectrum of compound (**3a**) gave a significant stable (M + 1) peak at *m/z* 213 which is also evident for the formation of compound (**3a**). Further, the spectrum of compound (**4a**), was considered as a representative example of the series (**4a–b)**. The IR spectrum showed the disappearance of the carbonyl stretching band of the ester group of compound (**3a**). The proton NMR spectrum of compound (**4a**) was established by the appearance of the OH stretching band at 3510–3600 cm^−1^, and the appearance of a broad singlet for the OH proton at δ 12.0 ppm and a decrease in one aromatic proton between 6.71 and 7.70 ppm. The mass spectrum of compound (**4a**) offered a significant stable (M + 1) peak at *m/z* 213 which is considered as additional evidence for the formation of this compound. Subsequently, compound (**5a**) was taken as a representative example for the **5(a–b)** series, which was confirmed by the appearance of the carbonyl stretching band for the ester group at 1760 cm^−1^ in the IR absorption spectrum. Moreover, it was confirmed by the disappearance of a broad singlet peak for the OH proton of compound (**4a**) and the appearance of a triplet and quartet for CH_3_ and CH_2_ protons at δ 2.31 and 4.15 ppm, respectively by proton NMR observation. Furthermore, the mass spectrum gave a significant stable (M + 1) peak at *m/z* 299 which clearly confirmed the formation of the compound (**5a**). The synthesis of compound (**6a**) was confirmed by the appearance of NH and NH_2_ stretching bands in the range between 3120–3220 cm^−1^ and carbonyl stretching band of amide at 1670 cm^−1^ in the IR spectrum. It was also confirmed by the proton NMR spectrum with the appearance of singlet amide −NH peak at δ 9.55 ppm and singlet NH_2_ peak around δ ppm and by the disappearance of triplet and quartet peaks for CH_3_ and CH_2_ protons respectively of compound (**5a**). The mass spectrum of this compound gave a significant stable (M + 1) peak at *m/z* 286 which also affirmed the formation of product (**6a**). Likewise, the structure of the compound (**8a**) was confirmed by the disappearance of NH_2_ band of compound (**6a**) and the appearance of C = N stretching band at 1630 cm^−1^ in the IR spectrum. Furthermore, in proton NMR, the appearance of a singlet peak of HC = N proton at δ 8.45 ppm, and an increase in four aromatic protons, confirmed the formation of the product (**8a**). The mass spectrum of compound (**8a**) gave two significant stable (M+) peak at *m/z* 407 and (M + 2) peak at 409 which also proves the formation of the compound (**8a**). Finally, the spectrum of the compound (**9a**) was considered as a representative example for the title compounds series **9(a–n)**. This was supported by the disappearance of C = N stretching band of the compound (**8a**) and by the appearance of the carbonyl stretching band of azetidinone ring at 1655 cm^−1^ in the IR spectrum. It was also proved by the NMR spectrum by the disappearance of singlet proton peak of HC = N and appearance of N-CH proton singlet peak at δ 5.45 ppm and singlet peak of Cl-CH at 5.6 ppm. The mass spectrum of compound (**9a**) gave two significant peaks of *m/z* 483 (M+) and 485 (M + 2), which also revealed the formation of the compound (**9a**).

### 3.3. Biology

#### In Vitro Antibacterial and Antifungal Activity

The development of hybrid drugs offers better treatment for various diseases, especially for microbial infections. Hybrid molecules with two or more pharmacophores have the potential to overcome drug resistance and reduce the risk of side effects through multiple mechanisms [59,60] and such compounds may inhibit two or more conventional targets simultaneously. It has been reported that molecules having heterocyclic moiety exhibited a wide range of biological activities. Therefore, hybridization of benzophenone moiety with 2-azetidinone heterocyclic pharmacophore increases the ability of new drug candidates that can resolve drug resistance problems. Considering these facts, a series of novel benzophenone fused azetidinone derivatives **9(a–n)** were efficiently synthesized with a moderate to good yield.

All the synthesized compounds **9(a–n)** were screened for antibacterial as well as antifungal activities. The antimicrobial activity was determined by using the disc diffusion method by means of measuring the zone of inhibition in mm, which was followed by the determination of Minimum Inhibitory Concentration (MIC), Minimum Bactericidal Concentration (MBC) and Minimum Fungicidal Concentration (MFC) of compounds **9a, 9e** and **9g** by broth dilution method against selected strains. In the series of compounds **9(a–n)**, some of them demonstrated activities ranging from good, moderate, to poor activity that has been summarized in Table 1 for antibacterial activity and in Table 2 for antifungal activity. Compounds **9a** with chloro group at the ortho position in phenyl ring, **9e** with the nitro group at the ortho position and chloro group at the meta position in the phenyl ring and **9g** with bromo group at meta position in the benzoyl ring exhibited maximum zone of inhibition in both bacterial and fungal strains compared that of the remaining analogues in the series with respect to standard drug *Chloramphenicol/Amoxicillin* in the case of bacterial strain (*B. subtilis, S. aureus, K. pneumoniae* and *P. aeruginosa)* and ketoconazole with respect to fungal strain (*A. niger* and *P. chrysogenum)*.

**Figure 4:**
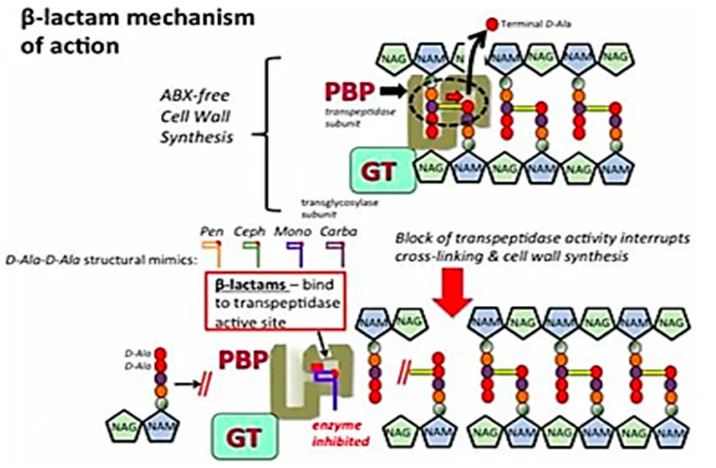
Mechanism of microbial growth inhibition by compounds **9e** and **9g**.

After the preliminary screening compounds **9a**, **9e** and **9g** were evolved as lead molecules. These lead molecules were again subjected to the broth dilution method to calculate the MIC, MBC, and MFC (μg/mL) for the selected strains. Among the lead molecules, compound **9e** with the nitro group at the ortho position and chloro group at the meta position in the phenyl ring and compound **9g** with bromo group at meta position in the benzoyl ring exhibited good inhibition at lower concentration against tested strains in comparison with that of the other analogues in the series. Compound **9a** with chloro group at the ortho position in phenyl ring, showed good antibacterial and moderate antifungal activity. Compounds **9j** and **9l** showed moderate antibacterial and antifungal activity, whereas compounds **9c**, **9d, 9h, 9i, 9k, 9m**, and **9n** showed less activity and compounds **9b** and **9f** with methoxy substituent showed no activity. In conclusion, the chloro and nitro substituents at the meta and ortho position in the phenyl ring have highly influenced the structure and morphology of the compound **9e** and the bromo substituent at the para position in the benzoyl ring of the compound **9g** shows the synergic effect, which in turn, is expected to be the reason for the inhibition of microbial growth. This is most likely due to the interaction of the β-lactam ring of compounds **9e** and **9g,** which gives the compound a three-dimensional shape that mimics the D-Ala-D-Ala peptide terminus that serves as the natural substrate for transpeptidase activity during cell wall peptidoglycan synthesis. Tight binding of these β-lactam drugs to the transpeptidase active site inhibits cell wall synthesis. Death results from osmotic instability caused by faulty cell wall synthesis, or the binding of the beta-lactam to penicillin binding proteins may trigger a series of events that lead to autolysis and death of the cell. Further, the diffusion of compounds **9e** and **9g** inside the cell membrane may also result in the ruin of vital functions of the cells such as replication, transcription, and translation. This may lead to DNA damage and imbalance in cell metabolism. (Figure 4). Finally, we can conclude that halo (chloro and bromo) and nitro substitutes have revealed good activity as seen in Table 3. Further, the in-vitro assay results were also compared with in-silico studies.

### 3.4. Molecular Docking Simulation

**Figure 5:**
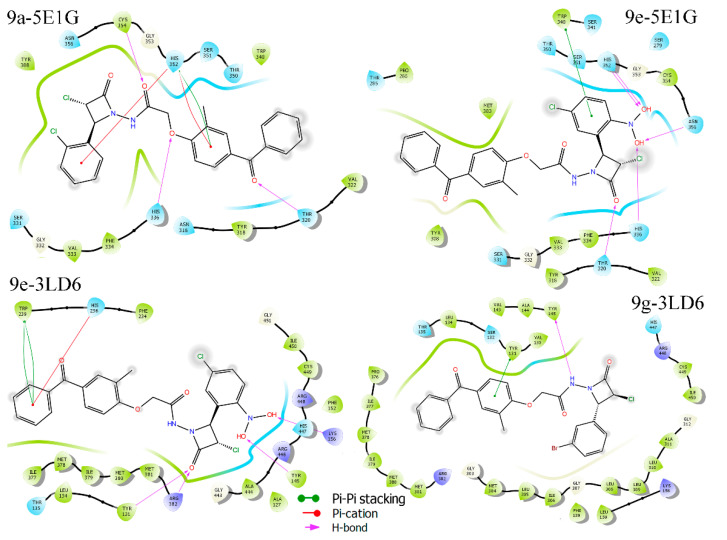
2D interaction plots of the ligands at the active site of the proteins showing pi- cation and hydrogen bond interactions.

The prediction of antibacterial and antifungal activities of the compounds have been carried out using in silico docking studies against (pdb ID: 5E1G) and (pdb ID: 3LD6) which are identified as a target for antibacterial and antifungal compounds, respectively [61,62]. Among all compounds, the outcomes of the current docking studies revealed the reasonable hydrogen bonds and binding affinity score for **9a, 9e**, and **9g** compounds that exhibited the best free energy and rational bonding interactions with the proteins and bridged diverse hydrogen bonds with the most important amino acids in the active site pockets of the proteins. The best binding energy value of **9a** compound against 5E1G protein found to be −8.99 kcal/mol for three hydrogen bonds with ligand efficiency and inhibition constant values of −0.27 and 2.14 µM, respectively (see Table 4). In this conformation, THR320 residue built a hydrogen bond with the oxygen atom of the carbonyl group attached to the bridge between phenyl and phenoxy rings at distance of 1.84 Å. Furthermore, the amino acids CYS354 and HIS336 also formed two hydrogen bonds with two oxygen atoms (=O and –O–) in acetamide bridge at distances of 1.95 and2.42 Å, respectively. Moreover, HIS352 residue exhibited two pi-cation and pi–pi stacking interactions with phenoxy ring as well as another one pi-cation interaction with chlorophenyl ring as depicted in Figure 5. The other docking conformations of **9a** with 5E1G protein also showed good results (Table 4).

The compound **9e** exhibited remarkable results with both proteins and recorded strong binding energies reaching up to −11.57 kcal/mol with 3LD6 protein through formation of four hydrogen bonds having ligand efficiency and inhibition constant values of −0.32 and 3.31 µM, respectively (see Table 4). The shortest hydrogen bond in this conformation has been formed between the residue LYS156N and one of the oxygen atoms attached to the nitro group at a distance of 1.67 Å, while another oxygen atom attached to the nitro group exhibited a hydrogen bond with the residue TYR145 at a distance of 1.98 Å. Furthermore, TYR131 and ARG382 residues formed two hydrogen bonds with the oxygen atom attached to the azetidine moiety at distances of 1.80 and 2.47 Å, respectively. Moreover, the phenyl ring in this conformation showed two pi–pi stacking interactions with TRP239 and also pi-cation interaction with HIS236 residue, respectively, (see Figure 5). The details of other conformations of **9e** compound with 3LD6 protein are listed in Table 4. On the other hand, the result of **9e** with 5E1G showed multiple effectiveness hydrogen bonds and affinity score, in which the best binding energy registered −9.86 kcal/mol associated with five hydrogen bonds and −0.27 and 59.52 µM values of ligand efficiency and inhibition constant, respectively, as listed in Table 4. In this conformation, one of oxygen atom attached to the nitro group built double hydrogen bonds with HIS352 amino acid at distances of 1.80 and 2.60 Å, respectively, and another oxygen atom attached to the nitro group constructed two hydrogen bonds with HIS336, and ANS356 residues at distances of 2.14 and 2.30 Å, respectively. Moreover, the oxygen atom attached to the azetidine moiety has formed the shortest hydrogen bond with THR320 residues at distance of 1.69 Å. Furthermore, this conformation is stabilized with pi–pi stacking interaction between the centroid of chlorophenyl ring and TRP340 amino acid as depicted in Figure 5. All other conformation of 9**e** compound docked with 5E1G protein are listed in Table 4.

Regarding to the docking result of **9g** compound with 3LD6 protein, the best conformation had −0.31 and 27.82 µM values of ligand efficiency and inhibition constant, respectively, with binding energy value of −10.31 kcal/mol for one hydrogen bond formed between TYR145 residue and nitrogen atom in the acetamide group at distance of 2.04 Å. Additionally, there is one pi–pi stacking interaction linked TYR131 residue with the centroid of phenoxy ring (see Figure 5). The parameters of all other docking conformations of **9g** compound with 3LD6 are listed in Table 4.

Hence, the synthesized compounds **9a** and **9e** fit nicely in the pocket site of 5E1G protein and are enclosed by several hydrophobic, hydrogens and pi contacts with the active amino residues TYR308, TYR318, THR320, GLY332, VAL333, PHE334, HIS336, TRP340, SER351, HIS352, GLY353, CYS354, ASN356. which found to be similar to the interaction seen in the cocrystal ligand and others 5E1G inhibitors structures [61]. On the other side, the synthesized compounds 9e and 9g also fit properly in 3LD6 active site and are surrounded by several hydrogen bonds, hydrophobic contacts, and pi interactions with the active amino acids TYR131, LEU134, TYR145, THR135, PHE152, LYS156, HIS236, TRP239, LIE377, MET380, MET381, ARG382, HIS447, CYS449, MET487, which are analogous to the interactions of original inhibitor of 3LD6 protein [62].

**Figure 6:**
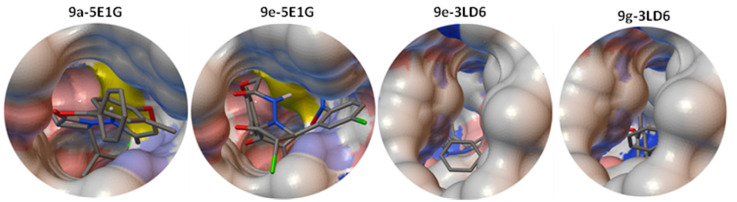
3-D close view visualization of the ligand-protein complexes showing the enfolding f the ligands in the active site groove of the proteins.

**Figure 7:**
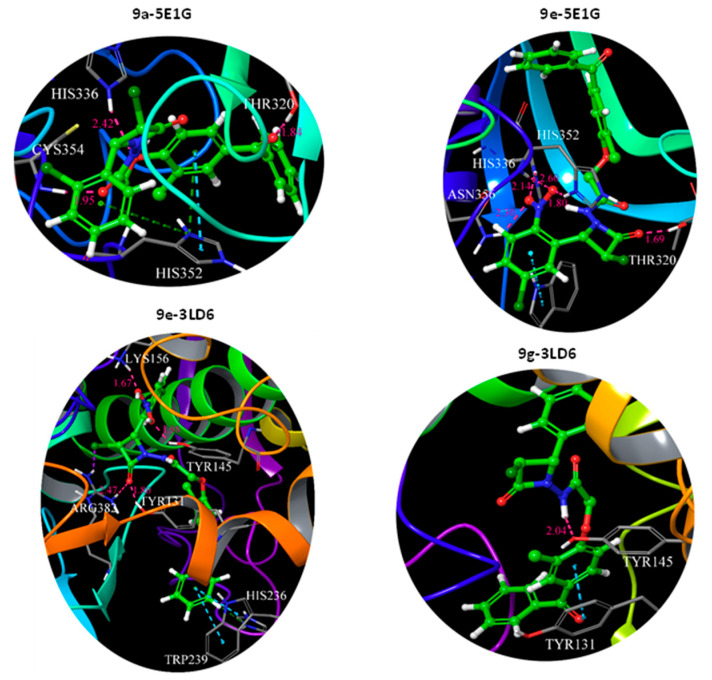
Ball and stick representation of the ligands with ribbon model of the protein targets showing hydrogen bond as dashed lines.

Figure 6 represents three-dimensional illustrations of the ligand-protein complexes with a close view showing the placing of the ligands in the active site groove of the proteins. The ribbon model of the protein targets with ligands in ball-stick representation for the best conformations are depicted in Figure 7.

**Figure 8:**
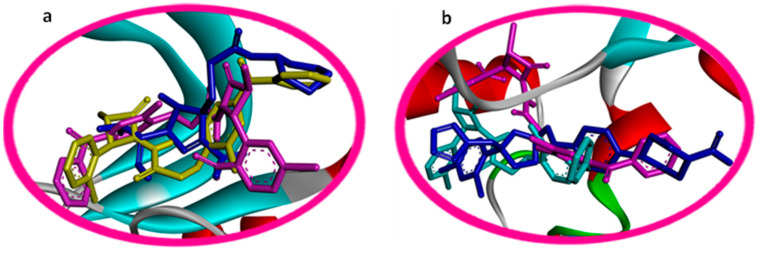
Overlapping of cocrystal ligands (blue color) with our synthesized compounds, **9a** (yellow color), **9e** (purple color) and **9g** (cyan color) docked with; (**a**) 5E1G and (**b**) 3LD6 proteins.

The docking simulations have been validated by redocking the original inhibitors, co-crystal ligands, and with the same proteins that exhibited good overlapping with our ligands having RMSD values of 0.172 and 0.201 for 9a and 9e docked with 5E1G (see Figure 8a). While RMSD values of **9e** and **9g** docked with 3LD6 are found to be 0.182 and 0.216, respectively, as illustrated in Figure 8b.

The result of in silico docking has been matched to the experimental result and revealed the importance of acetamide group, azetidinone, and phenyl ring in the biological activity of these compounds as antibacterial and antifungal candidates.

## 4. Conclusions

In conclusion, the synthesis of various benzophenone fused azetidinone derivatives were achieved by multi-step synthesis. All the synthesized compounds were characterized for structural confirmation. Further, newly synthesized benzophenone fused azetidinone derivatives were assessed for antibacterial and fungal activities. In vitro results revealed that the compounds **9a**, **9e**, and **9g** showed good antibacterial and antifungal activity. The remaining compounds demonstrated moderate to poor antimicrobial inhibition towards all the tested strains. On the other hand, in silico docking result has been matched the experimental results and revealed the importance of acetamide group, azetidinone, and phenyl ring in the biological activity of these compounds as antibacterial and antifungal candidates.

## Figures and Tables

**Table 1:** The in vitro antibacterial activity of compounds **9(a–n)**.

Compounds	Diameter of Zone of Inhibition (mm)															
	Gram-Positive Bacteria								Gram-Negative Bacteria							
	*B. subtilis*				*S. aureus*				*K. pneumoniae*				*P. aeruginosa*			
	12.5 μg/well	25 μg/well	50 μg/well	100 μg/well	12.5 μg/well	25 μg/well	50 μg/well	100 μg/well	12.5 μg/well	25 μg/well	50 μg/well	100 μg/well	12.5 μg/well	25 μg/well	50 μg/well	100 μg/well
**9a**	23 ± 2	23 ± 1	27 ± 3	29 ± 2	17 ± 1	18 ± 2	20 ± 1	23 ± 3	30 ± 1	31 ± 2	33 ± 3	36 ± 1	26 ± 2	29 ± 3	32 ± 1	33 ± 2
**9b**	-	-	-	-	-	-	-	-	-	-	-	-	-	-	-	-
**9c**	-	7 ± 3	9 ± 2	11 ± 3	-	-	-	-	12 ± 2	13 ± 1	15 ± 3	18 ± 1	9 ± 2	11 ± 2	14 ± 2	18 ± 2
**9d**	-	-	7 ± 1	9 ± 3	-	-	-	-	10 ± 2	11 ± 2	13 ± 2	16 ± 1	10 ± 2	12 ± 2	14 ± 2	17 ± 2
**9e**	22 ± 3	24 ± 1	27 ± 2	31 ± 1	16 ± 2	18 ± 3	20 ± 1	23 ± 2	31 ± 3	33 ± 1	35 ± 2	38 ± 1	28 ± 2	30 ± 1	33 ± 2	34 ± 3
**9f**	-	-	-	-	-	-	-	-	-	-	-	-	-	-	-	-
**9g**	21 ± 2	21 ± 1	25 ± 2	28 ± 3	13 ± 1	15 ± 3	19 ± 2	21 ± 3	29 ± 1	28 ± 3	31 ± 2	35 ± 1	25 ± 3	28 ± 2	30 ± 1	33 ± 1
**9h**	17 ± 1	19 ± 2	21 ± 1	24 ± 3	11 ± 2	13 ± 1	15 ± 2	17 ± 1	24 ± 2	26 ± 1	28 ± 1	31 ± 2	19 ± 1	23 ± 2	25 ± 2	29 ± 3
**9i**	9 ± 1	10 ± 3	11 ± 2	13 ± 1	-	-	-	8 ± 2	14 ± 1	18 ± 3	20 ± 1	21 ± 2	10 ± 2	12 ± 2	15 ± 1	19 ± 3
**9j**	15 ± 1	16 ± 2	18 ± 3	20 ± 1	9 ± 3	10 ± 2	12 ± 1	14 ± 2	21 ± 2	23 ± 1	25 ± 2	28 ± 1	16 ± 1	20 ± 2	22 ± 3	25 ± 2
**9k**	10 ± 2	13 ± 3	15 ± 1	17 ± 1	8 ± 3	9 ± 1	11 ± 2	12 ± 1	17 ± 1	19 ± 3	22 ± 1	24 ± 2	14 ± 2	16 ± 2	19 ± 1	23 ± 3
**9l**	16 ± 2	18 ± 3	20 ± 1	24 ± 2	10 ± 2	11 ± 1	13 ± 3	15 ± 1	22 ± 2	24 ± 2	26 ± 1	29 ± 2	18 ± 2	21 ± 1	24 ± 2	27 ± 3
**9m**	9 ± 1	11 ± 2	13 ± 3	15 ± 2	-	7 ± 1	8 ± 3	10 ± 1	16 ± 2	17 ± 1	19 ± 3	21 ± 2	11 ± 2	14 ± 1	16 ± 3	21 ± 1
**9n**	11 ± 2	13 ± 1	15 ± 2	18 ± 1	10 ± 1	11 ± 2	12 ± 1	13 ± 2	20 ± 3	21 ± 2	23 ± 1	26 ± 1	15 ± 1	19 ± 2	20 ± 2	24 ± 1
*Chloramphenicol*	33 ± 2	34 ± 2	36 ± 3	40 ± 1	30 ± 1	32 ± 3	35 ± 2	37 ± 3	38 ± 1	40 ± 2	42 ± 1	44 ± 1	25 ± 2	27 ± 3	29 ± 1	32 ± 2
*Amoxicillin*	30 ± 2	36 ± 1	28 ± 2	30 ± 2	32 ± 2	34 ± 1	32 ± 1	32 ± 2	35 ± 1	42 ± 1	40 ± 2	45 ± 1	28 ± 1	29 ± 2	28 ± 2	36 ± 1
Control (DMSO)	-	-	-	-	-	-	-	-	-	-	-	-	-	-	-	-

(-) No activity; (±) Standard deviation.

**Table 2:** The in vitro antifungal activity of compounds 9(a-n).

Compounds	Diameter of Zone of Inhibition (mm)							
	*A. niger*				*P. chrysogenum*			
	12.5 μg/well	25 μg/well	50 μg/well	100 μg/well	12.5 μg/well	25 μg/well	50 μg/well	100 μg/well
**9a**	25 ± 2	27 ± 2	29 ± 1	32 ± 3	18 ± 1	19 ± 2	20 ± 2	23 ± 3
**9b**	-	-	-	-	-	-	-	-
**9c**	11 ± 2	12 ± 2	14 ± 2	17 ± 2	-	-	8 ± 2	10 ± 2
**9d**	18 ± 3	20 ± 1	22 ± 1	25 ± 3	12 ± 2	14 ± 2	15 ± 1	17 ± 2
**9e**	32 ± 1	34 ± 3	36 ± 2	39 ± 2	23 ± 1	25 ± 3	27 ± 1	31 ± 2
**9f**	-	11 ± 2	8 ± 2	10 ± 2	-	-	-	-
**9g**	31 ± 2	33 ± 1	35 ± 1	38 ± 3	22 ± 2	23 ± 1	25 ± 2	29 ± 3
**9h**	22 ± 1	24 ± 1	27 ± 2	30 ± 1	13 ± 2	15 ± 3	17 ± 1	20 ± 2
**9i**	10 ± 2	11 ± 2	12 ± 2	15 ± 1	-	-	-	-
**9j**	27 ± 1	29 ± 3	31 ± 2	34 ± 2	17 ± 1	19 ± 3	21 ± 1	24 ± 2
**9k**	18 ± 2	19 ± 2	21 ± 3	24 ± 1	11 ± 2	12 ± 2	14 ± 2	16 ± 2
**9l**	26 ± 1	29 ± 2	32 ± 1	35 ± 2	20 ± 2	22 ± 1	24 ± 3	26 ± 2
**9m**	21 ± 1	22 ± 1	24 ± 3	27 ± 1	12 ± 2	14 ± 3	16 ± 1	19 ± 2
**9n**	15 ± 2	17 ± 1	19 ± 3	22 ± 2	9 ± 2	10 ± 2	12 ± 2	14 ± 3
Ketoconazole	30 ± 1	32 ± 3	34 ± 2	37 ± 3	35 ± 3	36 ± 1	37 ± 2	38 ± 3
Control (DMSO)	-	-	-	-	-	-	-	-

(-) No activity; (±) Standard deviation.

**Table 3:** MIC, MBC and MFC of compounds **9a, 9e**, and **9g**.

Compounds		Minimum Inhibitory Concentration, MIC, MBC and MFC g/well					
		*B. subtilis*	*S. aureus*	*K. pneumoniae*	*P. aeruginosa*	*A. niger*	*P. chrysogenum*
**9a**	MIC	25	50	12.5	6.25	12.5	50
	MBC	100	200	50	12.5	-	-
	MFC	-	-	-	-	50	200
**9e**	MIC	25	50	12.5	6.25	12.5	50
	MBC	100	200	50	12.5	-	-
	MFC	-	-	-	-	50	200
**9g**	MIC	50	100	25	25	6.25	25
	MBC	200	>200	100	100	-	-
	MFC	-	-	-	-	12.5	100
Chloramphenicol	MIC	6.25	6.25	12.5	6.25	-	-
Amoxicillin	MIC	12.5	12.5	6.25	6.25		
Ketoconazole	MIC					6.25	12.5

**Table 4:** Results of in silico docking studies for 9a, 9e and 9g ligands with 3U2K, antibacterial, and 1JIP, antifungal, targets.

Conf No.	Ligand	Protein	B.E (kcal/mole)	L. E	I.C,µM T = 298.15 K	vdW-Hb-Des-Energy kcal/mol	Hb of Residues and Ligands with Bond Length (Å)	Pi Interactions (Å)		* RMSD
								*pi–pi*	*pi*-Cation	
1	**9a**	5E1G	−8.99	−0.27	2.14	−10.99	THR320OH:O(1.84) CYS354NH:O(1.95) HIS336NH:O (2.42)	HIS352*–Cg2* (3.92)	HIS352*–Cg1* (3.00) HIS352*–Cg2* (2.68)	0.172
	**9e**		−9.86	−0.27	59.52	−1198	THR320 OH:O (1.69) HIS352NH:O (1.80) HIS352NH:O (2.60) HIS336NH:O (2.14) ANS356 NH:O (2.30)	TRP340*–Cg1* (3.59)		0.201
		3LD6	−11.57	−0.32	3.31	−12.07	LYS156NH:O (1.67) TYR131OH:O (1.80) TYR145O:HN (1.98) ARG382NH:O (2.47)	TRP239*–Cg3* (3.91) TRP239*–Cg3* (3.96)	HIS236*–Cg3* (2.84)	0.182
	**9g**		−10.31	−0.31	27.82	−12.42	TYR145O:HN (2.04)	TYR131*–Cg2* (3.92)		0.216
2	**9a**	5E1G	−8.67	−0.26	444.19	−10.69	THR320OH:O (2.10)		TRP340*–Cg1* (2.50)	1.046
	**9e**		−9.81	−0.27	64.39	−11.84	THR320OH:O (2.09) HIS352NH:O (1.82) HIS352NH:O (2.17)	TYR318*–Cg2* (4.00)	TRP340*–Cg1* (2.23)	1.311
		3LD6	−11.31	−0.31	5.16	−12.26	LYS156NH:O (2.24) HIS447NH:O (2.23) TYR145OH:O (1.91)			1.692
	**9g**		−10.06	−0.30	42.13	−12.18	HIS489NH:O (1.83)			25.669
3	**9a**	5E1G	−8.52	−0.26	566.15	−10.58				10.364
	**9e**		−9.57	−0.27	96.45	−11.91	THR320OH:O (1.69) HIS352NH:O (1.76)	TYR318*–Cg2* (3.84)	TRP340*–Cg1* (2.41)	2.504
		3LD6	−11.27	−0.31	5.52	−12.04	LYS156NH:O (1.90) HIS447NH:O (2.07) TYR145OH:O (2.02)			0.703
	**9g**		−9.42	−0.29	124.28	−11.41	LYS156NH:O (2.05)			0.958
4	**9a**	5E1G	−8.51	−0.26	580.09	−10.57	ASN356NH:O (2.14)		CYS354*–Cg2* (3.00)	8.259
	**9e**		−8.07	−0.22	1.21	−10.69	ASN356NH:O (1.78)		THR320*–Cg1* (2.72)	0.951
		3LD6	−10.90	−0.30	10.19	−12.35		PHE234*–Cg3* (4.00)	LYS156*–Cg1* (2.83)	4.989
	**9g**		−9.38	−0.30	62.65	−11.89				1.392
5	**9a**	5E1G	−8.05	−0.24	1.26	−10.09			TRP340*–Cg1* (2.88) HIS352*–Cg1* (2.96)	3.343
	**9e**		−7.19	−0.20	5.39	−9.73				13.219
		3LD6	−10.59	−0.29	17.39	−12.08		LYS156*–Cg1* (2.95)		3.821
	**9g**		−9.32	−0.28	148.35	−11.32	ILE379NH:O (2.11)	TRP239*–Cg3* (4.00)		
6	**9a**	5E1G	−8.03	−0.24	1.30	−10.05	THR320OH:O (2.23)			0.649
	**9e**		−6.71	−0.19	11.99	−9.23	THR320OH:O (1.93)		TYR318*–Cg2* (3.73)	0.826
		3LD6	−9.35	−0.26	140.29	−10.47	TYR145OH:O (1.56)			0.816
	**9g**		−9.30	−0.28	151.46	−11.41				5.911
7	**9a**	5E1G	−7.74	−0.23	256.69	−9.76	THR320OH:O (2.97)		TRP340*–Cg1* (2.36)	0.638
	**9e**		−5.67	−0.16	69.43	−8.19	TYR318OH:O (1.90)		HIS352*–Cg1* (2.74) HIS352*–Cg2* (2.83) TRP340*–Cg2* (2.93)	0.417
		3LD6	−9.00	−0.25	254.09	−11.06				8.952
	**9g**		−8.87	−0.27	316.81	−10.95	ILE450NH:O (2.01)			19.951
8	**9a**	5E1G	−6.87	−0.21	9.2	−8.96	THR320OH:O (2.17)			0.924
	**9e**		−5.43	−0.15	104.17	−7.92				15.240
		3LD6	−9.05	−0.25	231.56	−10.86	LYS156NH:O (2.16)			2.964
	**9g**		−8.76	−0.27	379.07	−10.82			LEU310*–Cg3* (2.86)	3.585
9	**9a**	5E1G	−6.00	−0.18	39.80	−7.97	HIS352NH:O (1.67) ASN356NH:O (1.67) HIS352NH:O (1.88)	TRP340*–Cg2* (4.00)	TYR318*–Cg1* (2.95)	0.258
	**9e**		−5.15	−0.14	168.76	−7.18	TPR340NH:O (1.63) ASN356NH:O (1.52)		HIS352*–Cg1* (2.57) HIS352*–Cg1* (2.59)	11.381
		3LD6	−7.90	−0.22	1.61	−10.16				14.948
	**9g**		−8.73	−0.26	396.42	−10.81				0.671
10	**9a**	5E1G	−5.86	−0.18	50.95	−7.95	HIS352NH:O (1.19) ASN356NH:O (1.66)			4.503
	**9e**		−4.20	−0.12	832.99	−6.74	THR320OH:O (1.59)			7.214
		3LD6	−6.61	−0.18	14.28	−7.40	LYS160NH:O (2.23) LYS436NH:O (1.97) ARG448NH:O (1.96)		GLY445*–Cg1* (2.41)	34.125
	**9g**		−7.66	−0.23	2.43	−9.78			ILE450*–Cg4* (2.92)	0.794

Conf. No.: Conformation Number, I.C: Inhibition Constant, B.E: Binding Energy, L.E: Ligand Efficiency, T: Temperature, vdW: Vander Walls energy, Hb: Hydrogen bond energy and Des: Desolv energy, *Cg1*, *Cg2*, *Cg3* and *Cg4* are centroids of chlorophyl, phenoxy, phenyl and bromophenyl rings, respectively. * RMSD values of our ligands and the original inhibitors, co-crystal ligands, redocked with the same proteins.

## Data Availability

All the data originated from this research is available from the authors under request.

## Supplementary Materials

The following supporting information can be downloaded at: https://www.mdpi.com/article/10.3390/cimb45010007/s1.

## References

[1] Prashanth T., Ranganatha V.L., Ramu R., Mandal S.P., Mallikarjunaswamy C., Khanum S.A. (2021). Synthesis, Characterization, Docking and Antimicrobial activity of 2-(4-benzoylphenoxy)-1-(2-((1-methyl-1H-indol-3-yl) methyl)-1H-benzo[d]imidazol-1-yl) ethanone derivatives. J. Iran. Chem. Soc..

[2] Khanum S.A., Shashikanth S., Umesha S., Kavitha R. (2005). Synthesis and Antimicrobial Study of Novel Heterocyclic Compounds from Hydroxy benzophenones. Eur. J. Med. Chem..

[3] Khanum S.A., Shashikanth S., Sudha B.S. (2003). A Facile Synthesis and Antimicrobial Activity of 3-(2-Aroylaryloxy) methyl-5-Mercapto-4-Phenyl-4H-1,2,4-Triazole and 2-(2-Aroylaryloxy) methyl-5-N-Phenylamino-1,3,4-Thiadiazole Analogues. Sci. Asia.

[4] CDC (2019). Antibiotic Resistance Threats in the United States, 2019.

[5] Francis J.S., Doherty M.C., Lopatin U., Johnston C.P., Sinha G., Ross T., Cai M., Hanse N.N., Per T., Ticehurst J.R. (2005). Severe community-onset pneumonia in healthy adults caused by methicillin-resistant Staphylococcus aureus carrying the Panton-Valentine leukocidin genes. Clin. Infect. Dis..

[6] Kruszewska D., Sahl H.G., Bierbaum G., Pag U., Hynes S.O., Ljungh A. (2004). Mersacidin eradicates methicillin-resistant Staphylococcus aureus MRSA in a mouse rhinitis model. Antimicrob. J. Chemother..

[7] Heel R.C., Brogden R.N., Carmine A., Morley P.A., Speight T.M., Avery G.S. (1982). Econazole: A review of its antifungal activity and therapeutic efficacy. Drugs.

[8] Sheehan D.J., Hitchcock C.A., Sibley C.M. (1999). Current and Emerging Azole Antifungal Agents. Clin. Microbiol. Rev..

[9] Lyman C.A., Walsh T.J. (1992). Systemically Administered Antifungal Agents: A Review of Their Clinical Pharmacology and Therapeutic Applications. Drugs.

[10] Clancy C.J., Nguyen M.H. (1998). In vitro efficacy and fungicidal activity of voriconazole against Aspergillus and Fusarium species. Eur. J. Clin. Microbiol. Infect. Dis..

[11] Fung-Tomc J.C., Huczko E., Minassian B., Bonner D.P. (1998). In Vitro Activity of a New Oral Triazole, BMS-207147 (ER-30346). Antimicrob. Agents Chemother..

[12] Espinel-Ingroff A. (1998). Comparison of In Vitro Activities of the New Triazole SCH56592 and the Echinocandins MK-0991 (L-743,872) and LY303366 against Opportunistic Filamentous and Dimorphic Fungi and Yeasts. J. Clin. Microbiol..

[13] Khadri M.J.N., Begum A.B., Sunil M.K., Khanum S.A. (2020). Synthesis, docking and biological evaluation of thiadiazole and oxadiazole derivatives as antimicrobial and antioxidant agents. Results Chem..

[14] Holden K.G., Morin R.B., Gorman M. (1982). Chemistry and Biology of b-Lactam Antibiotics.

[15] Mata E.G., Fraga M.A., Delpiccolo C.M.L. (2003). An Efficient, Stereoselective Solid-Phase Synthesis of β-Lactams Using Mukaiyama’s Salt for the Staudinger Reaction. J. Comb. Chem..

[16] Pawar R.P., Andurkar N.M., Vibhute Y.B. (1999). Studies on synthesis and antibacterial activity of some new Schiff bases, 4-thiazolidinones and 2-azetidinones. J. Indian Chem. Soc..

[17] Gootz T.D. (1990). Discovery and development of new antimicrobial agents. Clin. Microbiol. Rev..

[18] Maiti S.N. (2006). Overcoming bacterial resistance: Role of β-lactamase inhibitors. Top. Heterocycl. Chem..

[19] Singh G.S. (2004). Beta-lactams in the new millennium. Part-I: Monobactams and carbapenems. Mini-Rev. Med. Chem..

[20] Singh G.S. (2004). Beta-lactams in the new millennium. Part-II: Cephems, oxacephems, penams and sulbactam. Mini-Rev. Med. Chem..

[21] Risi C.D., Pollini G.P., Veronese A.C., Bertolasi V. (1999). A new simple route for the synthesis of (±)-2-azetidinones starting from β-Enaminoketoesters. Tetrahedron Lett..

[22] Georg G.I. (1993). The Organic Chemistry of b-Lactams.

[23] Abdulla R.F., Fuhr K.H. (1975). Monocyclic antibiotic beta-lactams. J. Med. Chem..

[24] Durckheimer W., Blumbach J., Lattrell R., Scheunemann K.H. (1985). Recent developments in the field of b-lactam antibiotics. Angew. Chem. Int. Ed. Engl..

[25] Kumar A., Rajput C.S., Bhati S.K. (2007). Synthesis of 3-[4′-(p-chlorophenyl)-thiazol-2′-yl]-2-[(substituted azetidinone/thiazolidinone)-aminomethyl]-6-bromoquinazolin-4-ones as anti-inflammatory agent. Bioorg. Med. Chem..

[26] Khanum S.A., Shashikanth S., Sudha B.S. (2004). Microwave-Assisted Synthesis of 2-Amino and 2-Azetidinonyl 5-(2-Benzoyl-phenoxymethyl)-1,3,4-Oxadiazoles. Het. Atom. Chem..

[27] Khanum S.A., Shashikanth S., Sathyanarayana S.G., Lokesh S., Deepak S.A. (2009). Synthesis and antifungal activity of 2-azetidinonyl-5-(2-benzoylphenoxy)methyl-1,3,4-oxadiazoles against seed-borne pathogens of *Eleusine coracana* (L.) Gaertn. Pest. Manag..

[28] Banik B.K., Becker F.F., Banik I. (2004). Synthesis of anticancer beta-lactams: Mechanism of action. Bioorg. Med. Chem..

[29] Gerona-Navarro G., de Vega M.J.P., Garcia-Lopez M.T., Andrei G., Snoeck R., de Clercq E., Balzarini J., Gonzalez-Muniz R. (2005). From 1-acyl-β-lactam human cytomegalovirus protease inhibitors to 1-benzyloxycarbonylazetidines with improved antiviral activity. A straightforward approach to convert covalent. J. Med. Chem..

[30] Yoakim C., Ogilvie W.W., Cameron D.R., Chabot C., Guse I., Haché B., Naud J., O’Meara J.A., Plante R., Dé ziel R. (1998). β-Lactam derivatives as inhibitors of human cytomegalovirus protease. J. Med. Chem..

[31] Alcaide B., Almendros P. (2002). Selective Bond Cleavage of the β-Lactam Nucleus: Application in Stereo controlled Synthesis. Synlett.

[32] Castagnolo D., Armaroli S., Corelli F., Botta M. (2004). Enantioselective synthesis of 1-aryl-2-propenylamines: A new approach to a stereoselective synthesis of the Taxol® side chain. Tetrahedron Asymmetry.

[33] Al-Ghorbani M., Thirusangu P., Gurupadaswamy H.D., Girish V. (2016). Synthesis and antiproliferative activity of benzophenone tagged pyridine analogues towards activation of caspase activated DNase mediated nuclear fragmentation in Dalton’s lymphoma. Bioorg. Chem..

[34] Neralagundi H.G.S., Begum A.B., Prabhakar B.T., Khanum S.A. (2016). Design and synthesis of diamide-coupled benzophenones as potential anticancer agents. Eur. J. Med. Chem..

[35] Al-Ghorbani M., Thirusangu P., Gurupadaswamy H.D., Vigneshwaran V. (2017). Synthesis of novel morpholine conjugated benzophenone analogues and evaluation of antagonistic role against neoplastic development. Bioorg. Chem..

[36] Gulnaz A.R., Mohammed Y.H.E., Khanum S.A. (2017). Design, synthesis, and molecular docking of benzophenone conjugated with oxadiazole sulphur bridge pyrazole pharmacophores as anti-inflammatory and analgesic agents. Bioorg. Chem..

[37] Eissa Mohammed Y.H., Gurupadaswamy H.D., Khanum S.A. (2017). Biological Evaluation of 2, 5-Di (4 Aryloylaryloxy Methyl)-1, 3, 4-Oxadiazoles Derivatives as Antimicrobial Agents. Med. Chem..

[38] Latha Rani N., Prashanth T., Zabiulla Sridhar M.A., Khanum S.A. (2016). Structural Study and Antibacterial Activity of a Benzophenone Derivative: [2-Bromo-4-(2-chloro-benzoyl)-phenoxy]-acetic acid ethyl ester. J. Appl. Chem..

[39] Azoro C. (2002). Antibacterial activity of crude extract of Azadiracta indica on Salmonella typhi. World J. Biotechnol..

[40] Chung K.T., Thomasson W.R., Wu-Yuan C.D. (1990). Growth inhibition of selected food-borne bacteria, particularly Listeria monocytogenes, by plant extracts. J. Appl. Bacteriol..

[41] Janovska D., Kubikova K., Kokoska L. (2003). Screening for antimicrobial activity of some medicinal plants species of traditional Chinese medicine. J. Food Sci..

[42] Bishnu J., Sunil L., Anuja S. (2009). Antibacterial Property of Different Medicinal Plants: Ocimum sanctum, Cinnamomum zeylanicum, Xanthoxylum armatum and Origanum majorana, Kathmandu university journal of science engineering and technology. J. Sci. Eng. Technol..

[43] Clinical and Laboratory Standards Institute (2006). Methods for Dilution Antimicrobial Susceptibility Tests for Bacteria That Grow Aerobically.

[44] Clinical and Laboratory Standards Institute (2002). Reference Method for Broth Dilution Antifungal Susceptibility Testing of Yeasts.

[45] Morris G.M., Huey R., Lindstrom W., Sanner M.F., Belew R.K., Goodsell D.S., Olson A.J. (2009). AutoDock4 and AutoDockTools4: Automated docking with selective receptor flexibility. J. Comput. Chem..

[46] Morris G.M., Goodsell D.S., Halliday R.S., Huey R., Hart W.E., Belew R.K., Olson A.J. (1998). Automated docking using a Lamarckian genetic algorithm and an empirical binding free energy function. J. Comput. Chem..

[47] Abad N., Sallam H.H., Al-Ostoot F.H., Khamees H.A., Al-horaibi S.A., Sridhar M.A., Khanum S.A., Madegowda M., El-hfi M., Maque J.T. (2021). Synthesis, crystal structure, DFT calculations, Hirshfeld surface analysis, energy frameworks, molecular dynamics and docking studies of novel isoxazolequinoxaline derivative (IZQ) as anti-cancer drug. J. Mol. Struct..

[48] Khamees H.A., Jyothi M., Khanum S.A., Madegowda M. (2018). Synthesis, crystal structure, spectroscopic characterization, docking simulation and density functional studies of 1-(3,4-dimethoxyphenyl)-3-(4-flurophenyl)-propan-1-one. J. Mol. Struct..

[49] Carr R.A., Congreve M., Murray C.W., Rees D.C. (2005). Fragment-based lead discovery: Leads by design. Drug Discov. Today.

[50] Shultz M.D. (2014). Improving the plausibility of success with inefficient metrics. ACS Med. Chem. Lett..

[51] Biovia D.S. Discovery Studio Visualiser v19.10.18287, 2018 (San Diego, CA, USA). https://www.ncbi.nlm.nih.gov/pmc/articles/PMC6770154/.

[52] (2015). The PyMOL Molecular Graphics System, 2015.

[53] Mehta P.D., Sengar N.P., Pathak A.K. (2010). 2-Azetidinone—A new profile of various pharmacological activities. Eur. J. Med. Chem..

[54] Bush K., Bradford P.A. (2016). β-Lactams and β-Lactamase Inhibitors: An Overview. Cold Spring Harb. Perspect. Med..

[55] Al-Ghorbani M., Lakshmi Ranganatha V., Prashanth T., Begum B., Khanum S.A. (2013). In vitro antibacterial and antifungal evaluation of some benzophenone analogues. Der Pharma Chem..

[56] Prashanth T., Naveen P., Al-Ghorbani M., Asha M.S., Khanum S.A. (2014). Synthesis and Inhibition of Microbial Growth by Benzophenone Analogues—A Simplistic Approach. Asian J. Biomed. Pharm. Sci..

[57] Lakshmi Ranganatha V., Khanum N.F., Khanum S.A. (2013). Synthesis, and evaluation of in vitro anti-microbial properties of novel benzophenone tagged indole analogues via 1, 3, 4-oxadiazole linkage. Int. J. Med. Pharm. Sci..

[58] Bushra Begum A., Khanum N.F., Lakshmi Ranganatha V., Prashanth T., Al-Ghorbani M., Khanum S.A. (2014). Evaluation of Benzophenone-N-ethyl Morpholine Ethers as Antibacterial and Antifungal activities. J. Chem..

[59] Meunier B. (2008). Hybrid molecules with a dual mode of action: Dream or reality?. Acc. Chem. Res..

[60] Bérubé G. (2016). An overview of molecular hybrids in drug discovery. Expert Opin. Drug Discov..

[61] Kumar P., Kaushik A., Lloyd E.P., Li S.G., Mattoo R., Ammerman N.C., Bell DT Perryman A.L., Zandi T.A., Ekins S., Ginell S.L. (2017). Non-classical transpeptidases yield insight into new antibacterials. Nat. Chem. Biol..

[62] Strushkevich N., Usanov S.A., Park H.W. (2010). Structural basis of human CYP51 inhibition by antifungal azoles. J. Mol. Biol..

