# Supplementary material for: Design, Synthesis, Characterization, and Analysis of Antimicrobial Property of Novel Benzophenone Fused Azetidinone Derivatives through In Vitro and In Silico Approach
Source: Curr Issues Mol Biol. 2022 Dec 23;45(1):92–109. doi: 10.3390/cimb45010007 (PMC9857151; doi:10.3390/cimb45010007)

Supplementary Material

Design, Synthesis, Characterization, and Analysis of Antimicrobial Property of Novel Benzophenone Fused Azetidinone Derivatives through In Vitro and In Silico Approach

Lakshmi Ranganatha Venkataravanappa [1], Mahima Jyothi [2], Hussien Ahmed Khamees [3] , Ekaterina Silina [4,5] ,Victor Stupin [4], Raghu Ram Achar [6,*] , Mohammed Al-Ghorbani [7,8] and Shaukath Ara Khanum [2,*]

1 Department of Chemistry, The National Institute of Engineering, Mysuru 570008, Karnataka, India
2 Department of Chemistry, Yuvaraja's College (Autonomous), University of Mysore, Mysuru 570005, Karnataka, India
3 Department of Medical Science, Community College-Abs, Hajjah ABS-00967, Yemen
4 Department of Surgery, Pirogov Russian National Research Medical University, 117997 Moscow, Russia
5 Institute of Biodesign and Modeling of Complex Systems, I.M. Sechenov First Moscow State Medical
University (Sechenov University), 119435 Moscow, Russia
6 Division of Biochemistry, School of Life Sciences, JSS Academy of Higher Education & Research, Mysuru 570015, Karnataka, India
7 Department of Chemistry, College of Science and Arts, Ulla, Taibah University, Medina 41477, Saudi Arabia
8 Department of Chemistry, College of Education, Thamar University, Thamar-425897, Yemen
* Correspondence: rracharya@jssuni.edu.in (R.R.A.); shaukathara@yahoo.co.in (S.A.K.)

2. Materials and Methods: Characterization Data

2.1. Chemistry

2.1.1. Characterization data for 3(a-b)

2-Methylphenyl benzoate 3a: Yield 90%, Pale yellow liquid. IR (Nujol): 1715 cm^{-1} (C=O). ^{1}H NMR (DMSO): δ 2.45 (s, 3H, Ar-CH_3), 7.0-7.8 (m, 9H, Ar-H). MS: *m/z* 213 (M+1). Anal. Calcd. For $C_{14}H_{12}O_2$ (212): C, 79.22; H, 5.70. Found: C, 79.18; H, 5.76%.

2-Methylphenyl-2-chlorobenzoate 3b: Yield 84%, Colour less liquid. IR (Nujol): 1710 cm^{-1} (C=O). ^{1}H NMR (DMSO): δ 2.33 (s, 3H, Ar-CH_3), 7.1-8.0 (m, 8H, Ar-H). MS: *m/z* 247 (M+), 249 (M+2). Anal. Calcd. For $C_{14}H_{11}ClO_2$ (247): C, 68.16; H, 4.49. Found: C, 68.19; H, 4.53%.

2.1.2. Characterization data for *4(a-b)*

(4-Hydroxy-3-methylphenyl)(phenyl)methanone 4a: Yield 72%, M.P. 110-112°C. IR (Nujol): 1640 (C=O), 3510-3600 cm^{-1} (OH). ^{1}H NMR (DMSO): δ 2.35 (s, 3H, CH_3), 6.71-7.70 (m, 8H, Ar-H), 13.0 (bs, 1H, -OH). MS: *m/z* 213 (M+1). Anal. Calcd. For $C_{14}H_{12}O_2$ (212.08): C, 79.22; H, 5.70. Found : C, 72.23; H, 5.69%.

(4-Hydroxy-3-methylphenyl)(2-chlorophenyl)methanone 4b: Yield 78%, M.P. 120-122°C. IR (Nujol): 1645 (C=O),3520-3650 cm^{-1} (OH). ^{1}H NMR (DMSO): δ 2.33 (s, 3H, CH_3), 6.73-7.71 (m, 7H, Ar-H), 12.10 (bs, 1H,

-OH). MS: *m/z* 246 (M+), 249 (M+2). Anal. Calcd. For $C_{14}H_{11}ClO_2$ (24): C, 68.16; H, 4.49. Found: C, 68.19; H, 4.53%.

2.1.3. Characterization data for *5(a-b)*

Ethyl 2-(4-benzoyl-2-methylphenoxy) acetate 5a: Yield 90%, M.P. 49-52°C. IR (Nujol): 1664 (C=O), 1760 cm^{-1} (ester, C=O). ^1H NMR (DMSO): δ 1.2 (t, 3H, CH_3 of ester), 2.3 (s, 3H, CH_3), 4.1 (q, 2H, CH_2 of ester), 4.5 (s, 2H, OCH_2), 7.1-7.7 (m, 8H, Ar-H). MS: *m/z* 299 (M+1). Anal. Calcd. For $C_{18}H_{18}O_4$ (298): C, 72.48; H, 6.04. Found: C, 72.46; H, 6.02%.

Ethyl 2-(4-(2-chlorobenzoyl)-2-methylphenoxy)acetate 5b: Yield 86%, M.P. 52-55°C. IR (Nujol): 1675 (C=O), 1755 cm^{-1} (ester, C=O). ^1H NMR (DMSO): δ 1.22 (t, 3H, CH_3 of ester), 2.4 (s, 3H, CH_3), 4.15 (q, 2H, CH_2 of ester), 4.6 (s, 2H, OCH_2), 6.9-7.8 (m, 7H, Ar-H). MS: *m/z* 333 (M+), 335 (M+2). Anal. Calcd. For $C_{18}H_{17}ClO_4$ (333): C, 64.97; H, 5.15. Found: C, 64.99; H, 5.19%.

2.1.4. Characterization data for *6(a-b)*

2-(4-Benzoyl-2-methylphenoxy)acetohydrazide 6a: Yield 80%, M.P. 125-128°C. IR (Nujol): 1630 (C=O), 1670 (amide,C=O), 3120-3220 cm^{-1} (NH-NH_2). ^1H NMR (DMSO): δ 2.2 (s, 3H, Ar-CH_3), 3.55 (bs, 2H, NH_2), 4.6 (s, 2H, CH_2), 7.2-7.8 (m, 8H, Ar-H), 9.35 (bs, 1H, CONH). MS: *m/z* 286 (M+1). Anal. Calcd. for $C_{16}H_{16}N_2O_3$ (285): C, 67.59; H, 5.67; N, 9.85. Found: C, 67.65; H, 5.74; N, 9.91%.

2-(4-(2-Chlorobenzoyl)-2-methylphenoxy)acetohydrazide 6b: Yield 85%, M.P. 140-142°C. IR (Nujol): 1636 (C=O), 1676 (amide,C=O),3115-3210 cm^{-1} (NH-NH_2). ^1H NMR (DMSO): δ 2.12 (s, 3H, Ar-CH_3), 3.45 (bs, 2H, NH_2), 4.62 (s, 2H, CH_2), 7.2-7.7 (m, 7H, Ar-H), 9.45 (bs, ^1H, CONH). MS: *m/z* 318 (M+), 320 (M+2). Anal. Calcd. for $C_{16}H_{15}ClN_2O_3$ (318): C, 60.29; H, 4.74; N, 8.79. Found: C, 60.32; H, 4.77; N, 8.83%.

2.1.5 Characterization data for *8(a-n)*

2-(4-Benzoyl-2-methylphenoxy)-N-(2-chlorobenzylidene) acetohydrazide 8a: Yield 80%, M.P. 170-172°C. IR (Nujol): 1715 (C=O), 1670 (amide,C=O), 3130-3210 (NH-N), 1630 cm^{-1} (N=CH). ^1H NMR (DMSO): δ 2.12 (s, 3H, Ar-CH_3), 4.6 (s, 2H, CH_2), 7.2-7.8 (m, 12H, Ar-H), 8.45 (s, 1H, N=CH), 9.55 (bs, 1H, CONH). MS: *m/z* 407 (M+), 409 (M+2). Anal. Calcd. for $C_{23}H_{19}ClN_2O_3$ (407): C, 67.90; H, 4.71; N, 6.89. Found: C, 67.94; H, 4.74; N, 6.92%.

2-(4-benzoyl-2-methylphenoxy)-N'-(4-methoxybenzylidene) acetohydrazide 8b: Yield 89%, M.P. 176-178°C. IR (Nujol): 1720 (C=O), 1675 (amide,C=O), 3130-3210 (NH-N), 1632 cm^{-1} (N=CH). ^1H NMR (DMSO): δ 2.12 (s, 3H, Ar-CH_3), 3.81 (s, 3H, Ar-OCH_3), 4.6 (s, 2H, CH_2), 7.2-7.8 (m, 12H, Ar-H), 8.45 (s, 1H, N=CH), 9.55 (bs, 1H, CONH). MS: *m/z* 403 (M+1). Anal. Calcd. for $C_{24}H_{22}N_2O_4$ (402): C, 71.63; H, 5.51; N, 6.96. Found: C, 71.64; H, 5.52; N, 6.95%.

2-(4-Benzoyl-2-methylphenoxy)-N-(4-hydroxybenzylidene) acetohydrazide 8c: Yield 82%, M.P. 182-185°C. IR (Nujol): 1720 (C=O), 1680 (amide,C=O), 3130-3210 (NH-N), 1635 cm^{-1} (N=CH). ^1H NMR (DMSO): δ 2.12 (s, 3H, Ar-CH_3), 4.6 (s, 2H, CH_2), 7.2-7.8 (m, 12H, Ar-H), 8.45 (s, 1H, N=CH), 9.55 (bs, 1H, CONH) 9.68 (s, 1H, Ar-OH). MS: *m/z*389 (M+1). Anal. Calcd. for $C_{23}H_{20}N_2O_4$ (388): C, 71.12; H, 5.19; N, 7.21. Found: C, 71.14; H, 5.19; N, 7.20%.

2-(4-Benzoyl-2-methylphenoxy)-N-(3-methylbenzylidene) acetohydrazide 8d:Yield 92%, M.P. 162-165°C. IR (Nujol): 1710 (C=O), 1675 (amide,C=O), 3130-3210 (NH-N), 1625 cm^{-1} (N=CH). ^{1}H NMR (DMSO): δ 2.12 (s, 3H, Ar-CH_3), 2.42 (s, 3H, Ar-CH_3), 4.6 (s, 2H, CH_2), 7.2-7.8 (m, 12H, Ar-H), 8.45 (s, 1H, N=CH), 9.55 (bs, 1H, CONH). MS: *m/z* 387 (M+1). Anal. Calcd. for $C_{24}H_{22}N_2O_3$ (386): C, 74.59; H, 5.74; N, 7.25. Found: C, 74.57; H, 5.74; N, 7.25%.

2-(4-Benzoyl-2-methylphenoxy)-'-(5-chloro-2-nitrobenzylidene) acetohydrazide 8e:Yield 89%, M.P. 192-194°C. IR (Nujol): 1715 (C=O), 1675 (amide,C=O), 3130-3210 (NH-N), 1625 cm^{-1} (N=CH). ^{1}H NMR (DMSO): δ 2.12 (s, 3H, Ar-CH_3), 4.6 (s, 2H, CH_2), 7.2-8.2 (m, 11H, Ar-H), 8.45 (s, 1H, N=CH), 9.55 (bs, 1H, CONH). MS: *m/z* 452 (M+), 454 (M+2). Anal. Calcd. for $C_{23}H_{18}ClN_3O_5$ (452): C, 61.14; H, 4.02; N, 9.30. Found: C, 61.13; H, 4.02; N, 9.28%.

2-(4-Benzoyl-2-methylphenoxy)-N-(2-methoxybenzylidene) acetohydrazide 8f: Yield 92%, M.P. 196-198°C. IR (Nujol): 1720 (C=O), 1672 (amide,C=O), 3130-3210 (NH-N), 1638 cm^{-1} (N=CH). ^{1}H NMR (DMSO): δ 2.12 (s, 3H, Ar-CH_3), 3.82 (s, 3H, Ar-OCH_3), 4.6 (s, 2H, CH_2), 7.2-8.2 (m, 12H, Ar-H), 8.45 (s, 1H, N=CH), 9.55 (bs, 1H, CONH). MS: *m/z* 403 (M+1) Anal. Calcd. for $C_{24}H_{22}N_2O_4$ (402): C, 71.63; H, 5.51; N, 6.96. Found: C, 71.61; H, 5.50; N, 6.95%.

2-(4-Benzoyl-2-methylphenoxy)-N-(3-bromobenzylidene) acetohydrazide 8g: Yield 92%, M.P. 184-186°C. IR (Nujol): 1730 (C=O), 1675 (amide,C=O), 3130-3210 (NH-N), 1625 cm^{-1} (N=CH). ^{1}H NMR (DMSO): δ 2.12 (s, 3H, Ar-CH_3), 4.6 (s, 2H, CH_2), 7.2-8.2 (m, 12H, Ar-H), 8.45 (s, 1H, N=CH), 9.55 (bs, 1H, CONH). MS: *m/z* 450 (M+), 452 (M+2). Anal. Calcd. for $C_{23}H_{19}BrN_2O_3$(450): C, 71.63; H, 5.51; N, 6.96. Found: C, 71.61; H, 5.50; N, 6.95%.

2-(4-(2-Chlorobenzoyl)-2-methylphenoxy)-N-(2-chlorobenzylidene) acetohydrazide 8h: Yield 85%, M.P. 160-163°C. IR (Nujol): 1730 (C=O), 1670 (amide,C=O), 3130-3210 (NH-N), 1635 cm^{-1} (N=CH). ^{1}H NMR (DMSO): δ 2.15 (s, 3H, Ar-CH_3), 4.5 (s, 2H, CH_2), 7.1-7.9 (m, 11H, Ar-H), 8.65 (s, 1H, N=CH), 9.35 (bs, 1H, CONH). MS: *m/z* 442 (M+), 443 (M+2). Anal. Calcd. for $C_{23}H_{18}Cl_2N_2O_3$ (442): C, 62.60; H, 4.11; N, 6.35. Found: C, 62.63; H, 4.14; N, 6.37%.

2-(4-(2-Chlorobenzoyl)-2-methylphenoxy)-N-(4-methoxybenzylidene) acetohydrazide 8i: Yield 88%, M.P. 176-178°C. IR (Nujol): 1735 (C=O), 1670 (amide,C=O), 3130-3210 (NH-N), 1655 cm^{-1} (N=CH). ^{1}H NMR (DMSO): δ 2.15 (s, 3H, Ar-CH_3), 4.5 (s, 2H, CH_2), 7.1-7.9 (m, 11H, Ar-H), 8.65 (s, 1H, N=CH), 9.35 (bs, 1H, CONH). MS: *m/z* 442 (M+), 444 (M+2). Anal. Calcd. for $C_{23}H_{18}Cl_2N_2O_3$ (442): C, 62.60; H, 4.11; N, 6.35. Found: C, 62.63; H, 4.14; N, 6.37%.

2-(4-(2-Chlorobenzoyl)-2-methylphenoxy)-N-(4-hydroxybenzylidene) acetohydrazide 8j: Yield 82%, M.P. 192-194°C. IR (Nujol): 1730 (C=O), 1675 (amide,C=O), 3130-3210 (NH-N), 1648 cm^{-1} (N=CH). ^{1}H NMR (DMSO): δ 2.15 (s, 3H, Ar-CH_3), 4.5 (s, 2H, CH_2), 7.1-7.9 (m, 11H, Ar-H), 8.65 (s, 1H, N=CH), 9.75 (bs, 1H, CONH) 9.98 (s, 1H, Ar-OH). MS: *m/z* 422 (M+), 424 (M+2). Anal. Calcd. for $C_{23}H_{19}ClN_2O_4$ (422): C, 65.33; H, 4.53; N, 6.62. Found: C, 65.32; H, 4.52; N, 6.61%.

2-(4-(2-Chlorobenzoyl)-2-methylphenoxy)-N-(3-methylbenzylidene) acetohydrazide 8k: Yield 87%, M.P. 179-181°C. IR (Nujol): 1732 (C=O), 1678 (amide,C=O), 3130-3210 (NH-N), 1655 cm^{-1} (N=CH). ^{1}H NMR (DMSO): δ 2.15 (s, 3H, Ar-CH_3), 2.42 (s, 3H, Ar-CH_3), 4.5 (s, 2H, CH_2), 7.1-7.9 (m, 11H, Ar-H), 8.65 (s, 1H, N=CH), 9.75 (bs, 1H,

CONH). MS: *m/z* 420 (M+), 422(M+2). Anal. Calcd. for $C_{24}H_{21}ClN_2O_3$ (420): C, 68.49; H, 5.03; N, 6.66. Found: C, 68.48; H, 5.02; N, 6.65%.

2-(4-(2-Chlorobenzoyl)-2-methylphenoxy)-N-(5-chloro-2-nitrobenzylidene) acetohydrazide 8l: Yield 89%, M.P. 192-194°C. IR (Nujol): 1740 (C=O), 1680 (amide,C=O), 3130-3210 (NH-N), 1655 cm^{-1} (N=CH). ^1H NMR (DMSO): δ 2.15 (s, 3H, Ar-CH_3), 4.5 (s, 2H, CH_2), 7.1-7.9 (m, 10H, Ar-H), 8.65 (s, 1H, N=CH), 9.35 (bs, 1H, CONH). MS: *m/z* 486 (M+), 488 (M+2). Anal. Calcd. for $C_{23}H_{17}Cl_2N_3O_5$ (486): C, 56.81; H, 3.52; N, 8.64. Found: C, 56.78; H, 3.52; N, 8.63%.

2-(4-(2-Chlorobenzoyl)-2-methylphenoxy)-N-(2-methoxybenzylidene) acetohydrazide 8m:Yield 89%, M.P. 182-185°C. IR (Nujol): 1735 (C=O), 1660 (amide,C=O), 3130-3210 (NH-N), 1655 cm^{-1} (N=CH). ^1H NMR (DMSO): δ 2.15 (s, 3H, Ar-CH_3), 3.42 (s, 3H, Ar-OCH_3), 4.5 (s, 2H, CH_2), 7.1-7.9 (m, 11H, Ar-H), 8.65 (s, 1H, N=CH), 9.75 (bs, 1H, CONH). MS: *m/z* 436 (M+), 438 (M+2). Anal. Calcd. for $C_{24}H_{21}ClN_2O_4$ (436): C, 65.98; H, 4.85; N, 6.41. Found: C, 65.97; H, 4.84; N, 6.%.

2-(4-(2-Chlorobenzoyl)-2-methylphenoxy)-N-(3-bromobenzylidene) acetohydrazide 8n: Yield 85%, M.P. 195-197°C. IR (Nujol): 1740 (C=O), 1675 (amide,C=O), 3130-3210 (NH-N), 1645 cm^{-1} (N=CH). ^1H NMR (DMSO): δ 2.15 (s, 3H, Ar-CH_3), 4.5 (s, 2H, CH_2), 7.1-7.9 (m, 11H, Ar-H), 8.65 (s, 1H, N=CH), 9.75 (bs, 1H, CONH). MS: *m/z* 484 (M+), 486 (M+2). Anal. Calcd. for $C_{23}H_{18}BrClN_2O_3$ (484): C, 56.87; H, 3.74; N, 5.77. Found: C, 56.86; H, 3.73; N, 5.76.%.

2.1.6. Characterization data for *(9a-n)*

2-(4-Benzoyl-2-methylphenoxy)-N-[3-chloro-4-(2-chlorophenyl)-2-oxoazetidin-1-yl] acetamide 9a: Yield 85%, M.P. 150-152°C. IR (Nujol): 1730 (C=O), 1655 cm^{-1} (N-CO), 1670 (amide,C=O), 3130-3210cm^{-1} (NH-N). ^1H NMR (DMSO): δ2.14 (s, 3H, Ar-CH_3), 4.6 (s, 2H, CH_2), 5.45 (s, 1H, N-CH), 5.6 (s, 1H, Cl-CH), 6.75-7.77 (m, 12H, Ar-H), 11.12 (s, 1H, CONH). ^{13}C NMR (DMSO): 17.4 (IC, Ar-CH_3), 62.3 (1C, Az-ring), 63.6 (1C, Az-Cl ring), 66.9 (1C, CH_2), 113.9 (1C, Ar-C), 124.2 (1C,Ar-C), 126.6 (1C, Ar-C), 128.1 (1C,Ar-C), 128.3 (2C, Ar-C), 128.4 (2C, Ar-C), 128.6 (1C, Ar-C), 130.3 (2C, Ar-C), 131.5 (1C, Ar-C), 131.9 (1C, Ar-C), 132.2 (1C, Ar-C), 132.4 (1C, Ar-C), 138.4 (1C, Ar-C), 143.5 (1C, Ar-C), 162.3 (1C, Ar-C),163.5 (1C, Az-C=O), 166.3 (1C, C=O amide), 194.3 (1C, Ar-C=O). MS: *m/z* 483 (M+), 485 (M+2). Anal. Calcd. for $C_{25}H_{20}Cl_2N_2O_4$ (483): C, 62.12; H, 4.17; N, 5.80. Found: C, 62.14; H, 4.15; N, 5.81%.

2-(4-Benzoyl-2-methylphenoxy)-N-[3-chloro-4-(4-methoxyphenyl)-2-oxoazetidin-1-yl] acetamide 9b: Yield74%, M.P. 162-165°C. IR (Nujol): 1735 (C=O), 1665 (amide,C=O), 1636 (N-CO), 3130-3215 cm^{-1} (NH-N).^1H NMR (DMSO): δ2.12 (s, 3H, Ar-CH_3), 4.2 (s, 3H, O-CH_3), 4.5 (s, 2H, CH_2), 5.46 (s, 1H, N-CH), 5.8 (s, 1H, Cl-CH), 6.77-7.76 (m, 12H, Ar-H), 9.56 (bs, 1H, CONH). ^{13}C NMR (DMSO): 16.3 (IC, Ar-CH_3), 55.8 (1C, Ar-OCH_3), 64.1 (1C, Az-ring), 66.9 (1C, Az-Cl ring), 67.4 (1C, CH_2), 113.9 (1C, Ar-C), 114.1 (2C, Ar-C), 124.2 (1C,Ar-C), 126.6 (2C, Ar-C), 128.1 (1C, Ar-C), 128.3 (1C, Ar-C), 128.4 (2C, Ar-C), 130.3 (2C, Ar-C), 131.5 (1C, Ar-C), 131.9 (1C, Ar-C), 132.4 (1C, Ar-C), 138.4 (1C, Ar-C), 143.5 (1C, Ar-C), 162.3 (1C, Ar-C), 163.5 (1C, Az-C=O), 166.3 (1C, C=O amide), 194.3 (1C, Ar-C=O).MS: *m/z* 478 (M+), 480 (M+2). Anal. Calcd. for $C_{26}H_{23}ClN_2O_5$ (478): C, 65.20; H, 4.84; N, 5.85. Found: C, 65.22; H, 4.86; N, 5.86. %.

2-(4-Benzoyl-2-methylphenoxy)-N-3-chloro-4-(4-hydroxyphenyl)-2-oxoazetidin-1-yl] acetamide 9c: Yield70%, M.P. 142-145°C. IR (Nujol): 1730 (C=O), 1668 (amide,C=O), 1636 (N-CO), 3130-3215 (NH-N)3530-3600

cm^{-1} (OH). ^{1}H NMR (DMSO): δ2.15 (s, 3H, Ar-CH_3), 4.5 (s, 2H, CH_2), 5.46 (s, 1H, N-CH), 5.8 (s, 1H, Cl-CH), 6.75-7.78 (m, 12H, Ar-H), 9.06 (S, 1H, Ar-OH), 9.54 (bs, 1H, CONH).^{13}C NMR (DMSO): 15.7 (IC, Ar-CH_3), 64.1 (1C, Az-ring), 66.9 (1C, Az-Cl ring), 67.3 (1C, CH_2), 113.7 (1C, Ar-C), 115.4 (2C, Ar-C), 124.2 (1C,Ar-C), 127.2 (2C, Ar-C), 128.1 (1C, Ar-C), 128.3 (1C, Ar-C), 128.4 (2C, Ar-C), 130.3 (2C, Ar-C), 131.5 (1C, Ar-C), 131.9 (1C, Ar-C), 132.4 (1C, Ar-C), 138.4 (1C, Ar-C), 143.5 (1C, Ar-C), 162.3 (1C, Ar-C), 163.3 (1C, Az-C=O), 166.3 (1C, C=O amide), 194.3 (1C, Ar-C=O). MS: *m/z* 464 (M+), 466 (M+2). Anal. Calcd. for $C_{25}H_{21}ClN_2O_5$ (464): C, 64.59; H, 4.55; N, 6.03. Found: C, 64.55; H, 4.57; N, 6.01%.

2-(4-Benzoyl-2-methylphenoxy)-N-[3-chloro-4-(3-methylphenyl)-2-oxoazetidin-1-yl] acetamide 9d: Yield78%, M.P. 152-155°C. IR (Nujol): 1730 (C=O), 1668 (amide,C=O), 1636 cm^{-1} (N-CO), 3130-3215 cm^{-1} (NH-N). ^{1}H NMR (DMSO): δ2.15 (s, 3H, Ar-CH_3), 2.21 (s, 3H, Ar-CH_3), 4.6 (s, 2H, CH_2), 5.48 (s, 1H, N-CH), 5.6 (s, 1H, Cl-CH), 6.71-7.76 (m, 12H, Ar-H), 9.55 (bs, 1H, CONH).).^{13}C NMR (DMSO): 15.6 (IC, Ar-CH_3), 21.6 (1C, Ar-CH_3), 64.1 (1C, Az-ring), 66.9 (1C, CH_2), 67.7 (1C, Az-Cl ring), 113.9 (1C, Ar-C), 123.9 (1C, Ar-C), 124.2 (1C,Ar-C), 126.7 (1C, Ar-C), 127.0 (1C, Ar-C), 128.0 (1C, Ar-C), 128.1 (1C, Ar-C), 128.3 (1C, Ar-C), 128.4 (2C, Ar-C), 130.3 (2C, Ar-C), 131.5 (1C, Ar-C), 131.9 (1C, Ar-C), 132.4 (1C, Ar-C), 138.2 (1C, Ar-C), 143.4 (1C, Ar-C), 162.3 (1C, Ar-C), 163.4 (1C, Az-C=O), 166.5 (1C, C=O amide), 193.3 (1C, Ar-C=O). MS: *m/z* 462 (M+), 464 (M+2). Anal. Calcd. for $C_{26}H_{23}ClN_2O_4$(462): C, 67.46; H, 5.01; N, 6.05. Found: C, 67.45; H, 5.04; N, 6.02%.

2-(4-Benzoyl-2-methylphenoxy)-N-[3-chloro-4-(5-chloro-2-nitrophenyl)-2-oxoazetidin-1-yl] acetamide 9e: Yield78%, M.P. 161-162°C. IR (Nujol): 1560 (NO_2), 1730 (C=O), 1668 (amide,C=O), 1636 cm^{-1} (N-CO), 3130-3215 cm^{-1} (NH-N). ^{1}H NMR (DMSO): δ2.12 (s, 3H, Ar-CH_3), 2.25 (s, 3H, Ar-CH_3), 4.4 (s, 2H, CH_2), 5.46 (s, 1H, N-CH), 5.5 (s, 1H, Cl-CH), 6.7-7.78 (m, 10H, Ar-H), 9.54 (bs, 1H, CONH). ^{13}C NMR (DMSO): 16.6 (IC, Ar-CH_3), 62.3 (1C, Az-ring), 63.4 (1C, Az-Cl ring), 66.7 (1C, CH_2), 113.9 (1C, Ar-C), 124.2 (1C,Ar-C), 126.4 (1C, Ar-C), 127.6 (1C, Ar-C), 127.7 (1C, Ar-C), 128.3 (1C, Ar-C), 128.4 (2C, Ar-C), 128.6 (1C, Ar-C), 130.3 (2C, Ar-C), 131.5 (1C, Ar-C), 131.9 (1C, Ar-C), 132.4 (1C, Ar-C), 138.4 (1C, Ar-C), 140.2 (1C, Ar-C), 145.3 (1C, Ar-C), 162.3 (1C, Ar-C), 163.5 (1C, Az-C=O), 166.3 (1C, C=O amide), 194.3 (1C, Ar-C=O). MS: *m/z*527 (M+), 529 (M+2). Anal. Calcd. for $C_{25}H_{19}Cl_2N_3O_6$(527): C, 56.82; H, 3.64; N, 7.95. Found: C, 56.84; H, 3.63; N, 7.93%.

2-(4-Benzoyl-2-methylphenoxy)-N-[3-chloro-4-(2-methoxyphenyl)-2-oxoazetidin-1-yl] acetamide 9f: Yield78%, M.P. 164-167°C. IR (Nujol): 1735 (C=O), 1665 (amide,C=O), 1636 (N-CO), 3130-3215 cm^{-1} (NH-N).^{1}H NMR (DMSO): δ2.12 (s, 3H, Ar-CH_3), 4.2 (s, 3H, O-CH_3), 4.5 (s, 2H, CH_2), 5.46 (s, 1H, N-CH), 5.8 (s, 1H, Cl-CH), 6.77-7.76 (m, 12H, Ar-H), 9.56 (bs, 1H, CONH).^{13}C NMR (DMSO): 15.9 (IC, Ar-CH_3), 56.2 (1C, Ar-CH_3), 61.5 (1C, Az-ring), 64.4 (1C, Az-Cl ring), 66.9 (1C, CH_2), 112.1 (1C, Ar-C), 113.9 (1C, Ar-C), 120.9 (1C, Ar-C), 124.2 (1C, Ar-C), 127.7 (1C, Ar-C), 128.1 (1C, Ar-C), 128.3 (1C, Ar-C), 128.4 (2C, Ar-C), 129.3 (1C, Ar-C), 130.3 (2C, Ar-C), 131.5 (1C, Ar-C), 131.9 (1C, Ar-C), 132.4 (1C, Ar-C), 138.4 (1C, Ar-C), 156.4 (1C, Ar-C), 162.3 (1C, Ar-C), 163.4 (1C, Az-C=O), 166.5 (1C, C=O amide), 193.3 (1C, Ar-C=O). MS: *m/z* 478 (M+), 480 (M+2). Anal. Calcd. for $C_{26}H_{23}ClN_2O_5$ (478): C, 65.20; H, 4.84; N, 5.85. Found: C, 65.22; H, 4.86; N, 5.86. %.

2-(4-Benzoyl-2-methylphenoxy)-N-[4-(3-bromophenyl)-3-chloro-2-oxoazetidin-1-yl] acetamide 9g: Yield 85%, M.P. 170-172°C. IR (Nujol):

1730 (C=O), 1655 cm^{-1} (N-CO), 1670 (amide,C=O), 3130-3210cm^{-1} (NH-N). ^{1}H NMR (DMSO): δ2.19 (s, 3H, Ar-CH_3), 4.2 (s, 2H, CH_2), 5.46 (s, 1H, N-CH), 5.7 (s, 1H, Cl-CH), 6.75-7.77 (m, 12H, Ar-H), 9.55 (bs, 1H, CONH).^{13}C NMR (DMSO): 15.8 (IC, Ar-CH_3), 64.1 (1C, Az-Cl ring), 66.7 (1C, Az-ring), 66.9 (1C, CH_2), 113.9 (1C, Ar-C), 122.9 (1C, Ar-C), 124.2 (1C, Ar-C), 125.9 (1C, Ar-C), 128.3 (1C, Ar-C), 128.4 (2C, Ar-C), 129.3 (1C, Ar-C), 129.6 (1C, Ar-C), 130.3 (2C, Ar-C), 131.5 (1C, Ar-C), 131.7 (1C, Ar-C), 131.9 (1C, Ar-C), 132.4 (1C, Ar-C), 138.4 (1C, Ar-C), 145.7 (1C, Ar-C), 162.3 (1C, Ar-C), 163.5 (1C, Az-C=O), 166.3 (1C, C=O amide), 194.3 (1C, Ar-C=O). MS: *m/z*526 (M+), 528 (M+2). Anal. Calcd. for $C_{25}H_{20}BrClN_2O_4$ (526): C, 56.89; H, 3.82; N, 5.31. Found: C, 56.87; H, 3.84; N, 5.33%.\

2-(4-(2-Chlorobenzoyl)-2-methylphenoxy)-N-[3-chloro-4-(2-chlorophenyl)-2-oxoazetidin-1-yl]acetamide 9h: Yield 85%, M.P. 150-152°C. IR (Nujol): 1730 (C=O), 1655 cm^{-1} (N-CO), 1670 (amide,C=O), 3130-3210cm^{-1} (NH-N). ^{1}H NMR (DMSO): δ2.14 (s, 3H, Ar-CH_3), 4.6 (s, 2H, CH_2), 5.45 (s, 1H, N-CH), 5.6 (s, 1H, Cl-CH), 6.75-7.77 (m, 11H, Ar-H), 9.55 (bs, 1H, CONH). ^{13}C NMR (DMSO): 15.6 (IC, Ar-CH_3), 62.3 (1C, Az-ring), 63.6 (1C, Az-Cl ring), 66.9 (1C, CH_2), 113.9 (1C, Ar-C), 124.2 (1C,Ar-C), 126.5 (1C, Ar-C), 126.6 (1C, Ar-C), 128.1 (1C, Ar-C), 128.3 (1C, Ar-C), 128.4 (1C, Ar-C), 128.5 (1C, Ar-C). 128.6 (1C, Ar-C), 131.5 (1C, Ar-C), 131.9 (1C, Ar-C), 132.2 (1C, Ar-C), 132.4 (1C, Ar-C), 133.8 (1C, Ar-C), 136.4 (1C, Ar-C), 139.3 (1C, Ar-C), 143.5 (1C, Ar-C), 162.3 (1C, Ar-C), 163.5 (1C, Az-C=O), 166.3 (1C, C=O amide), 194.3 (1C, Ar-C=O). MS: *m/z*517 (M+), 518 (M+2), 520 (M+4). Anal. Calcd. for $C_{25}H_{19}Cl_3N_2O_4$ (516): C, 57.99; H, 3.70; N, 5.41. Found: C, 57.97; H, 3.72; N, 5.43%.

2-(4-(2-Chlorobenzoyl)-2-methylphenoxy)-N-[3-chloro-4-(4-methoxyphenyl)-2-oxoazetidin-1-yl]acetamide 9i: Yield74%, M.P. 166-167°C. IR (Nujol): 1735 (C=O), 1665 (amide,C=O), 1636 (N-CO), 3130-3215 cm^{-1} (NH-N).^{1}H NMR (DMSO): δ2.12 (s, 3H, Ar-CH_3), 4.2 (s, 3H, O-CH_3), 4.5 (s, 2H, CH_2), 5.46 (s, 1H, N-CH), 5.8 (s, 1H, Cl-CH), 6.77-7.76 (m, 11H, Ar-H), 9.56 (bs, 1H, CONH).). ^{13}C NMR (DMSO): 16.4 (IC, Ar-CH_3), 55.8 (1C, Ar-OCH_3), 64.1 (1C,Az-Cl ring), 66.9 (1C, CH_2), 67.4 (1C, Az-ring), 113.9 (1C, Ar-C), 114.1 (2C, Ar-C), 124.2 (1C, Ar-C), 126.5 (1C, Ar-C), 126.6 (2C, Ar-C), 128.3 (1C, Ar-C), 128.5 (1C, Ar-C), 129.7 (1C, Ar-C), 131.5 (1C, Ar-C), 131.9 (1C, Ar-C), 133.8 (1C, Ar-C), 135.8(1C, Ar-C), 136.4 (1C, Ar-C), 139.3 (1C, Ar-C), 158.6 (1C, Ar-C), 162.3 (1C, Ar-C), 163.5 (1C, Az-C=O), 166.3 (1C, C=O amide), 194.3 (1C, Ar-C=O). MS: *m/z*512 (M+), 514 (M+2). Anal. Calcd. for $C_{26}H_{22}Cl_2N_2O_5$(512): C, 60.83; H, 4.32; N, 5.46. Found: C, 60.85; H, 4.35; N, 5.47%.

2-(4-(2-Chlorobenzoyl)-2-methylphenoxy)-N-[3-chloro-4-(4-hydroxyphenyl)-2-oxoazetidin-1-yl] acetamide 9j: Yield70%, M.P. 140-143°C. IR (Nujol): 1730 (C=O), 1668 (amide,C=O), 1636 (N-CO), 3130-3215 (NH-N)3530-3600 cm^{-1} (OH). ^{1}H NMR (DMSO): δ2.15 (s, 3H, Ar-CH_3), 4.5 (s, 2H, CH_2), 4.8 (bs, 1H, -OH), 5.46 (s, 1H, N-CH), 5.8 (s, 1H, Cl-CH), 6.75-7.78 (m, 11H, Ar-H), 9.54 (bs, 1H, CONH).^{13}C NMR (DMSO): 15.8 (1C, Ar-CH_3), 64.1 (1C,Az-Cl ring), 66.9 (1C, CH_2), 67.4 (1C, Az-ring), 113.9 (1C, Ar-C), 115.7 (2C, Ar-C), 124.2 (1C, Ar-C), 126.5 (1C, Ar-C), 127.2 (2C, Ar-C), 128.3 (1C, Ar-C), 128.5 (1C, Ar-C), 129.7 (1C, Ar-C), 131.5 (1C, Ar-C), 131.9 (1C, Ar-C), 133.8 (1C, Ar-C), 136.1 (1C, Ar-C), 136.4 (1C, Ar-C), 139.3 (1C, Ar-C), 156.5 (1C, Ar-C), 162.3 (1C, Ar-C), 163.5 (1C, Az-C=O), 166.3 (1C, C=O amide), 194.3 (1C, Ar-C=O). MS: *m/z* 464 (M+), 466 (M+2). Anal. Calcd. for $C_{25}H_{20}Cl_2N_2O_5$(464): C, 60.13; H, 4.04; N, 5.61. Found: C, 60.15; H, 4.06; N, 5.63%.

2-(4-(2-Chlorobenzoyl)-2-methylphenoxy)-N-[3-chloro-4-(3-methylphenyl)-2-oxoazetidin-1-yl]acetamide 9k: Yield78%, M.P. 152-155°C. IR (Nujol): 1730 (C=O), 1668 (amide,C=O), 1636 cm^{-1} (N-CO), 3130-3215 cm^{-1} (NH-N). ^{1}H NMR (DMSO): δ2.15 (s, 3H, Ar-CH_3), 2.21 (s, 3H, Ar-CH_3), 4.6 (s, 2H, CH_2), 5.48 (s, 1H, N-CH), 5.6 (s, 1H, Cl-CH), 6.71-7.76 (m, 11H, Ar-H), 9.55 (bs, 1H, CONH). ^{13}C NMR (DMSO): 15.7 (1C, Ar-CH_3), 21.5 (1C, Ar-C), 64.1 (1C,Az-Cl ring), 66.9 (1C, CH_2), 67.4 (1C, Az-ring), 113.9 (1C, Ar-C), 123.9 (1C, Ar-C), 124.2 (1C, Ar-C), 126.5 (1C, Ar-C), 126.7 (1C, Ar-C), 127.0 (1C, Ar-C), 127.2 (1C, Ar-C), 128.3 (1C, Ar-C), 128.5 (1C, Ar-C), 129.7 (1C, Ar-C), 131.5 (1C, Ar-C), 131.9 (1C, Ar-C), 133.8 (1C, Ar-C), 136.1 (1C, Ar-C), 136.4 (1C, Ar-C), 139.3 (1C, Ar-C), 156.5 (1C, Ar-C), 162.3 (1C, Ar-C), 163.5 (1C, Az-C=O), 166.3 (1C, C=O amide), 194.3 (1C, Ar-C=O). MS: *m/z* 496 (M+), 498 (M+2). Anal. Calcd. for $C_{26}H_{22}Cl_2N_2O_4$(496): C, 62.79; H, 4.46; N, 5.63. Found: C, 62.76; H, 4.44; N, 5.65%.

2-(4-(2-Chlorobenzoyl)-2-methylphenoxy)-N-[3-chloro-4-(5-chloro-2-nitrophenyl)-2-oxoazetidin -1-yl]acetamide 9l: Yield78%, M.P. 151-152 °C. IR (Nujol): 1560 (NO_2), 1730 (C=O), 1668 (amide,C=O), 1636 cm^{-1} (N-CO), 3130-3215 cm^{-1} (NH-N). ^{1}H NMR (DMSO): δ2.12 (s, 3H, Ar-CH_3), 2.25 (s, 3H, Ar-CH_3), 4.4 (s, 2H, CH_2), 5.46 (s, 1H, N-CH), 5.5 (s, 1H, Cl-CH), 6.7-7.78 (m, 9H, Ar-H), 9.54 (bs, 1H, CONH).^{13}C NMR (DMSO): 16.6 (1C, Ar-CH_3), 62.3 (1C, Az-ring), 63.4 (1C, Az-Cl ring), 66.9 (1C, CH_2), 113.9 (1C, Ar-C), 124.2 (1C, Ar-C), 124.3 (1C, Ar-C), 126.4 (1C, Ar-C), 126.5 (1C, Ar-C), 127.6 (1C, Ar-C), 127.7 (1C, Ar-C), 128.5 (1C, Ar-C), 129.7 (1C, Ar-C), 131.5 (1C, Ar-C), 131.9 (1C, Ar-C), 133.4 (1C, Ar-C), 136.4 (1C, Ar-C), 138.4 (1C, Ar-C), 139.3 (1C, Ar-C), 140.2 (1C, Ar-C), 145.3 (1C, Ar-C), 162.3 (1C, Ar-C), 163.5 (1C, Az-C=O), 166.3 (1C, C=O amide), 194.3 (1C, Ar-C=O). MS: *m/z*561 (M+), 563 (M+2). Anal. Calcd. for $C_{25}H_{18}Cl_3N_3O_6$(561): C, 53.35; H, 3.22; N, 7.47. Found: C, 53.36; H, 3.24; N, 7.46%.

2-(4-(2-Chlorobenzoyl)-2-methylphenoxy)-N–[3-chloro-4-(2-methoxyphenyl)-2-oxoazetidin -1-yl]acetamide 9m: Yield74%, M.P. 167-168°C. IR (Nujol): 1735 (C=O), 1665 (amide,C=O), 1636 (N-CO), 3130-3215 cm^{-1} (NH-N).^{1}H NMR (DMSO): δ2.12 (s, 3H, Ar-CH_3), 4.2 (s, 3H, O-CH_3), 4.5 (s, 2H, CH_2), 5.46 (s, 1H, N-CH), 5.8 (s, 1H, Cl-CH), 6.77-7.76 (m, 11H, Ar-H), 9.56 (bs, 1H, CONH). ^{13}C NMR (DMSO): 15.9 (IC, Ar-CH_3), 56.9 (1C, Ar-CH_3), 61.5 (1C, Az-ring), 64.4 (1C, Az-Cl ring), 66.9 (1C, CH_2), 112.1 (1C, Ar-C), 113.9 (1C, Ar-C), 120.9 (1C, Ar-C), 124.2 (1C, Ar-C), 126.1 (1C, Ar-C), 126.5 (1C, Ar-C), 127.7 (1C, Ar-C),128.3 (1C, Ar-C), 128.5 (1C, Ar-C), 129.3 (1C, Ar-C), 129.7 (1C, Ar-C), 131.5 (1C, Ar-C), 131.9 (1C, Ar-C), 133.8 (1C, Ar-C), 136.4 (1C, Ar-C), 139.4 (1C, Ar-C), 156.4 (1C, Ar-C), 162.3 (1C, Ar-C), 163.4 (1C, Az-C=O), 166.5 (1C, C=O amide), 193.3 (1C, Ar-C=O).MS: *m/z*512 (M+), 514 (M+2). Anal. Calcd. for $C_{26}H_{22}Cl_2N_2O_5$(512): C, 60.83; H, 4.32; N, 5.46. Found: C, 60.85; H, 4.35; N, 5.47%.

2-(4-(2-Chlorobenzoyl)-2-methylphenoxy)-N–[3-chloro-4-(3-bromophenyl)-2-oxoazetidin -1-yl]acetamide 9n: Yield 85%, M.P. 172-174°C. IR (Nujol): 1730 (C=O), 1655 cm^{-1} (N-CO), 1670 (amide,C=O), 3130-3210cm^{-1} (NH-N). ^{1}H NMR (DMSO): δ2.19 (s, 3H, Ar-CH_3), 4.2 (s, 2H, CH_2), 5.46 (s, 1H, N-CH), 5.7 (s, 1H, Cl-CH), 6.75-7.77 (m, 11H, Ar-H), 9.55 (bs, 1H, CONH). ^{13}C NMR (DMSO): 15.7 (IC, Ar-CH_3), 61.5 (1C, Az-ring), 64.4 (1C, Az-Cl ring), 66.9 (1C, CH_2), 112.1 (1C, Ar-C), 113.9 (1C, Ar-C), 122.9 (1C, Ar-C), 124.2 (1C, Ar-C), 125.9 (1C, Ar-C), 126.5 (1C, Ar-C), 128.3 (1C, Ar-C), 128.5 (1C, Ar-C), 129.3 (1C, Ar-C), 129.6 (1C, Ar-C), 129.7 (1C,

Ar-C), 131.5 (1C, Ar-C), 131.9 (1C, Ar-C), 133.8 (1C, Ar-C), 136.4 (1C, Ar-C), 139.4 (1C, Ar-C), 145.4 (1C, Ar-C), 162.3 (1C, Ar-C), 163.4 (1C, Az-C=O), 166.5 (1C, C=O amide), 193.3 (1C, Ar-C=O).MS: *m*/*z*560 (M+1), 561 (M+2). Anal. Calcd. for $C_{25}H_{19}BrCl_2N_2O_4$ (559): C, 53.41; H, 3.41; N, 4.98. Found: C, 53.45; H, 3.43; N, 4.95%.

Spectral Data of compound

^1H NMR for 2-Methylphenyl benzoate (3a)

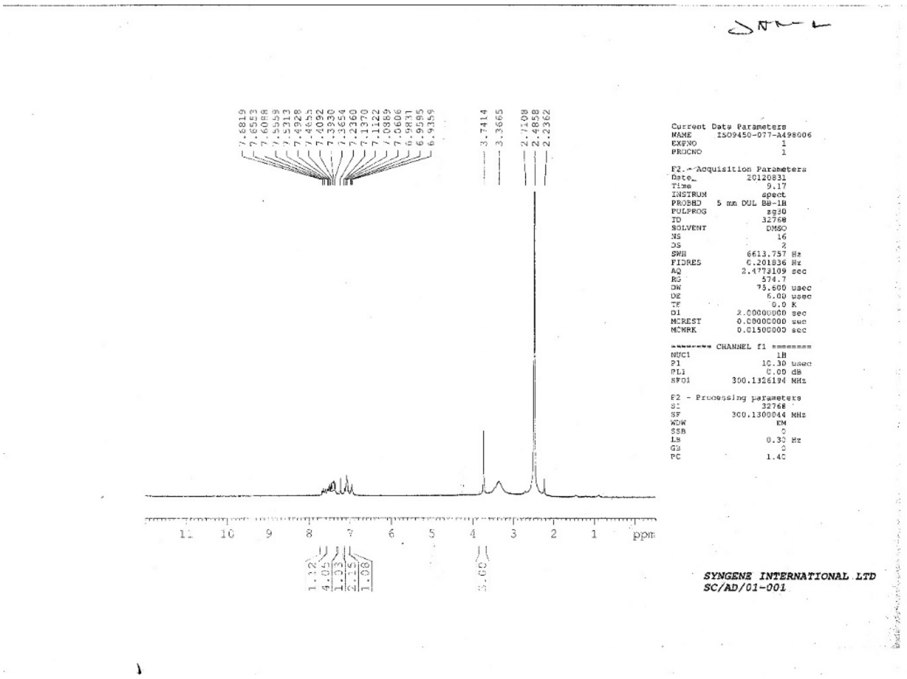

^{1}H NMR for (4-Hydroxy-3-methylphenyl)(phenyl)methanone (4a)

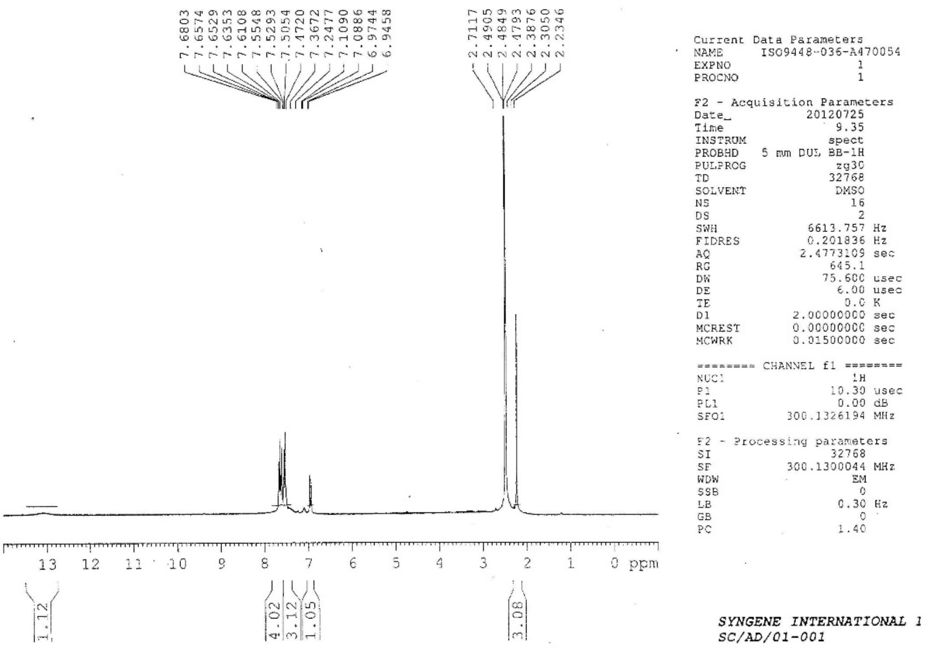

^{1}H NMR for Ethyl 2-(4-(2-chlorobenzoyl)-2-methylphenoxy)acetate (5b)

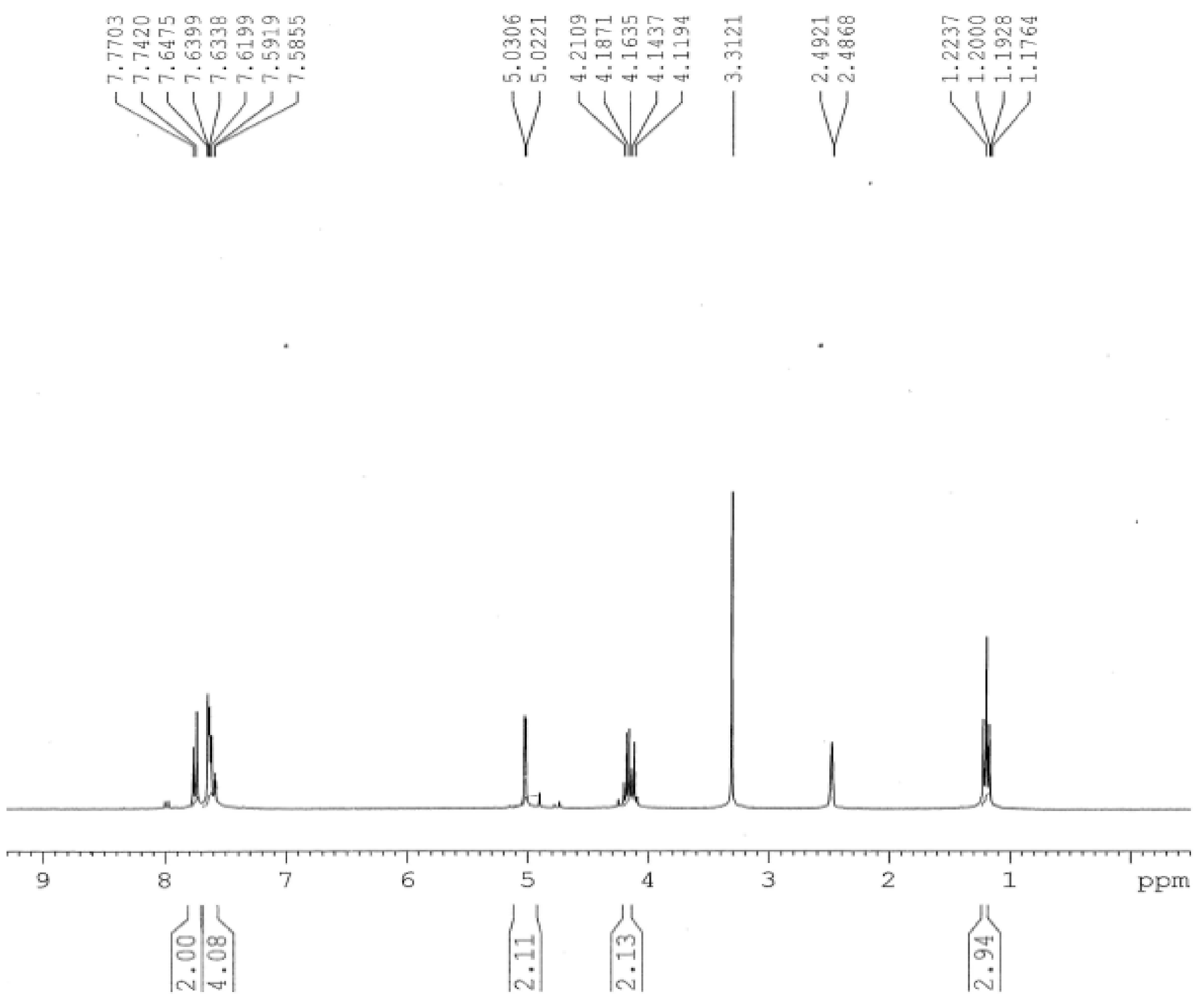

^{1}H NMR for 2-(4-Benzoyl-2-methylphenoxy)acetohydrazide (6a)

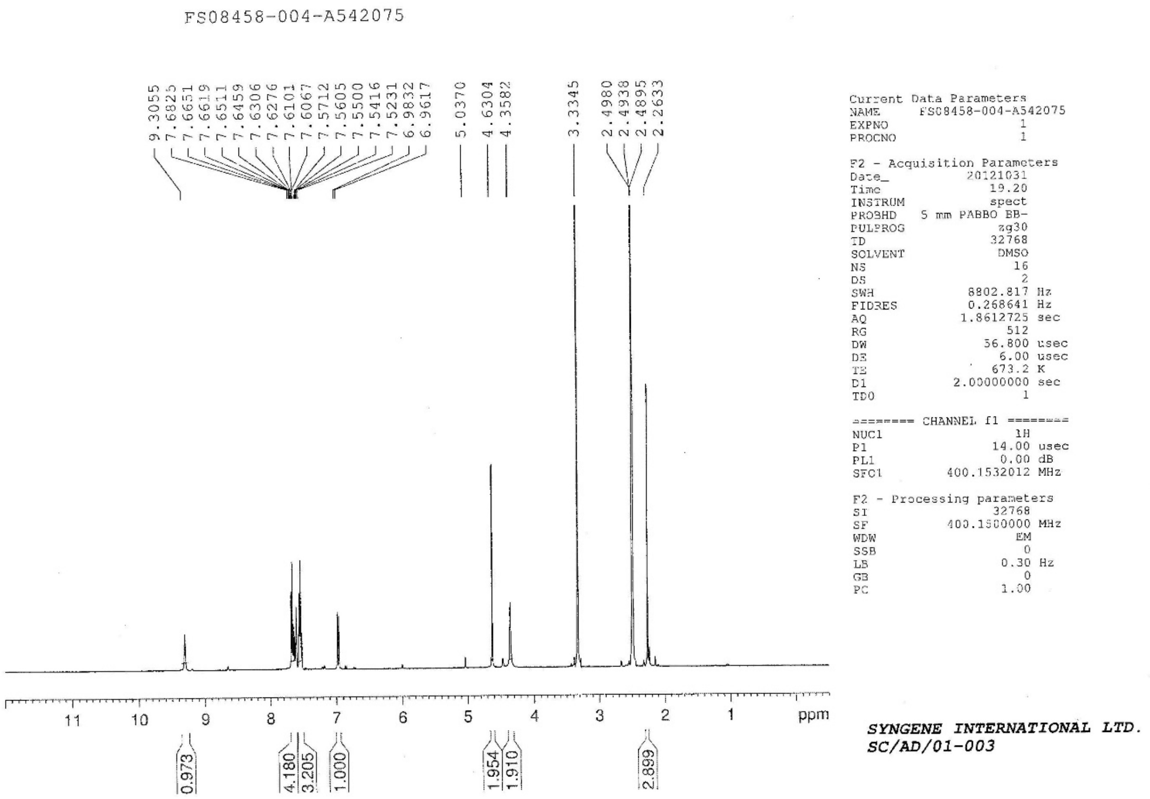

^{1}H NMR for 2-(4-Benzoyl-2-methylphenoxy)-N-(2-chlorobenzylidene)acetohydrazide (8a)

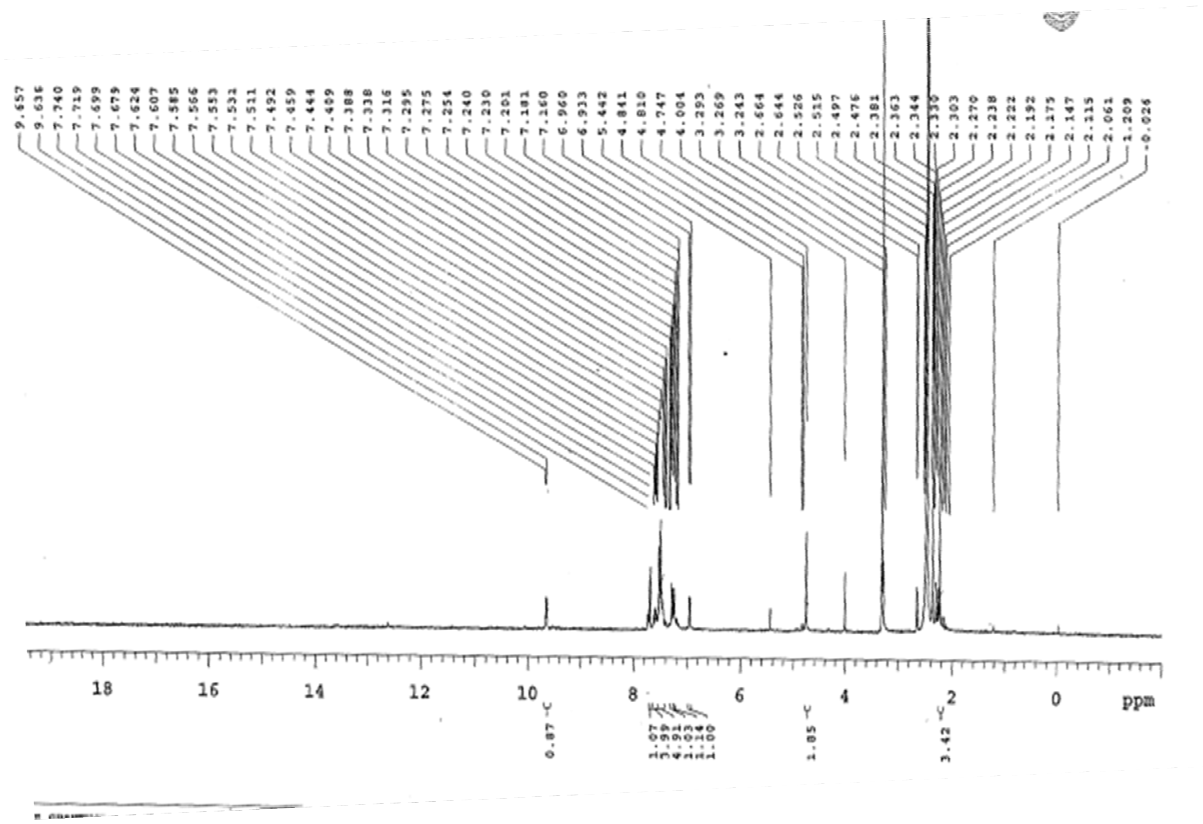

2-(4-Benzoyl-2-methylphenoxy)-N-[3-chloro-4-(2-chlorophenyl)-2-oxoazetidin-1-yl] acetamide (9a)

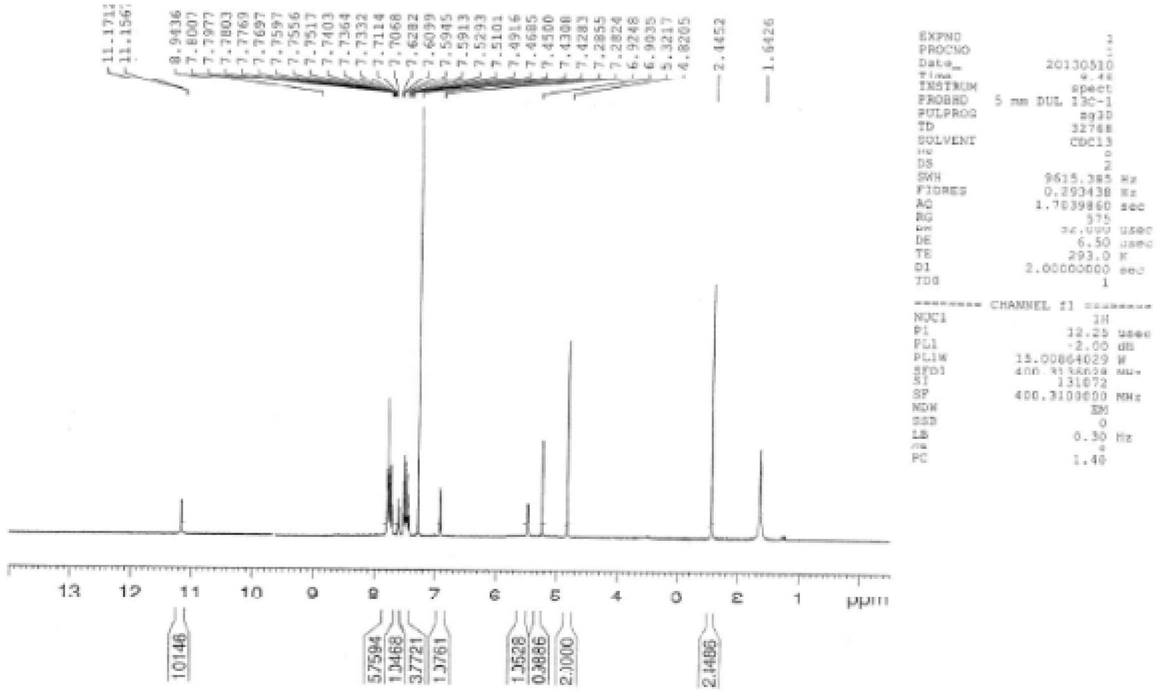

Supplement: Supplementary file 1 [file cimb-45-00007-s001.zip › cimb-1955095-supplementary.pdf]
